# Variability in cadmium tolerance of closely related *Listeria monocytogenes* isolates originating from dairy processing environments

**DOI:** 10.1128/aem.01281-24

**Published:** 2024-11-21

**Authors:** Andrea Domen, Jenna Porter, Jared Johnson, James Molyneux, Lorraine McIntyre, Jovana Kovacevic, Joy Waite-Cusic

**Affiliations:** 1 Food Innovation Center, Oregon State University 705130 , Portland, Oregon, USA; 2 Department of Food Science and Technology, Oregon State University 2694 , Corvallis, Oregon, USA; 3 Department of Statistics, Oregon State University 2694 , Corvallis, Oregon, USA; 4 BC Centre for Disease Control 113269 , Vancouver, British Columbia, Canada; The Pennsylvania State University, University Park, Pennsylvania, USA

**Keywords:** *Listeria monocytogenes*, cadmium tolerance, efflux pumps, dairy processing environment, whole genome sequencing

## Abstract

Increased tolerance to cadmium in *Listeria monocytogenes* has been suggested to contribute to their persistence in natural and food production environments. This study investigated the phenotypic cadmium response of *L. monocytogenes* strains with efflux pump *cadAC* (variants 1–4) and related strains with *cadA1C1*. Growth of *cadAC* variant strains (*n* = 5) in 0 µM–120 µM cadmium salts (CdCl_2_, CdSO_4_) in Mueller-Hinton broth (MHB) was evaluated. Additionally, 88 *L*. *monocytogenes* strains from dairy processing facilities were exposed to 43.8 µM CdCl_2_ in MHB, and their lag phase duration (LPD) was measured. Strains with *cadA1* through *cadA3* showed similar growth trends in the presence of cadmium, while the *cadA4* variant (Scott A) had the highest CdCl_2_ minimum inhibitory concentration (175 µM). Growth varied between the two salts, with CdSO_4_ significantly increasing LPD (*P* < 0.05) compared to CdCl_2_. In 43.8 µM CdCl_2_, *cadA1* strains displayed LPDs ranging from 0.99 ± 0.14 h to 6.44 ± 0.08 h, with no clear genomic differences explaining this variability. Strains without *cadA* did not grow at 43.8 µM CdCl_2_ but exhibited low tolerance (10.9 µM CdCl_2_), potentially due to non-specific soft metal ATPases (626 aa; 737 aa) and soft metal resistance proteins encoded by *czc* genes (289 aa; 291 aa; 303 aa) within their chromosomes. These findings enhance our understanding of *L. monocytogenes* cadmium tolerance and underscore the need for further research to explore the genetic and physiological factors underlying these trends.

**IMPORTANCE:**

Mobile genetic elements in *Listeria monocytogenes* contribute to its survival in natural and food processing environments. This study focused on how different genetic variants of the efflux pump gene *cadAC* and group of closely related *cadA1C1* strains respond to cadmium exposure. When exposed to two cadmium salts, cadmium chloride and cadmium sulfate, we observed varying growth patterns, with a significantly longer lag phase in cadmium sulfate compared to cadmium chloride. Strains with *cadA1* to *cadA3* had similar growth trends, whereas a strain with the *cadA4* variant had the highest minimum inhibitory concentration value. Among 88 strains from dairy processing facilities, significant phenotypic differences were observed despite core genome similarities, indicating other underlying genetic and physiological factors contribute to cadmium tolerance. Since cadmium tolerance studies in *L. monocytogenes* are limited, with rare phenotypic comparisons between closely related strains, our study makes an important observation and contribution to understanding of *L. monocytogenes* tolerance to cadmium by providing phenotypic comparisons between numerous strains within the same clonal group (<16 single nucleotide polymorphisms).

## INTRODUCTION

*Listeria monocytogenes* is a Gram-positive, facultative anaerobe with one of the highest mortality rates among the foodborne pathogens. Its ubiquity in natural and agricultural environments, as well as its psychrotrophic abilities, make this pathogen a particular challenge for the food industry. *L. monocytogenes* has shown an ability to persist in dairy processing environments, including cheese processing facilities, sometimes for years (1, 2). Typically, persistence is defined as the repeated isolation of identical subtypes of *L. monocytogenes* over an extended period of time (3). Subtyping classification of strains differs by study and may range from non-sequence-based methods, such as pulsed-field gel electrophoresis and serotyping, to sequence-based methods, such as single nucleotide polymorphisms (SNPs) (3, 4). While reasons behind the persistence of *L. monocytogenes* are typically complex and multifaceted, cadmium tolerance (minimum inhibitory concentration [MIC] ≥256 ppm) has been shown to be more common in resident strains in comparison to sporadic or transient strains from raw milk and non-dairy foods (5). This is hypothesized to be due to environmental cadmium contamination from anthropogenic sources, making it advantageous for *L. monocytogenes* to adopt these mechanisms (5, 6).

While cadmium can be naturally found alongside zinc ore in the earth’s crust, anthropogenic sources are responsible for much of the metal found in the environment (7, 8). These sources range from cadmium byproducts in inorganic phosphate fertilizer to mining, to burning coal (7, 8). Worldwide, there is between 0.01 and 1 mg/kg of cadmium in the soil, with an average of 0.36 mg/kg (8). Cadmium enters the cells through divalent cation channels (Mg^2+^, Mn^2+^, Zn^2+^) (9, 10). It is toxic to bacteria, as it binds to sulfhydryl groups of essential proteins (e.g., respiratory proteins) and generates free oxidative radicals (11, 12).

Bacteria, including *L. monocytogenes,* have two main strategies to manage intracellular heavy metal concentration: efflux pumps that actively remove heavy metal ions and small cysteine-rich proteins that sequester heavy metal ions (13). Cadmium efflux pump genes in *L. monocytogenes* are well documented, with the first cadmium efflux pump, *cadA1*, found on a plasmid-associated transposon Tn*5422* nearly 30 years ago (13). The second cadmium efflux pump, CadA2, was discovered on plasmid pLM80, and has similar lengths and aa homology to CadA1 (711 aa for CadA1 and 705 aa for CadA2; 69% identity) (14). Additional cadmium efflux pumps, CadA3 and CadA4, have been identified on the chromosome of some *L. monocytogenes* strains (15, 16), with CadA3 being quite rare (17). Notably, CadA4 has higher divergence compared to the first three cadmium efflux pumps and has been associated with lower cadmium resistance (18). Other efflux pump genes that act on heavy metals have been documented in *L. monocytogenes*, including *mdrL*; however, it has not been associated with cadmium tolerance (19).

All cadmium-specific efflux pump operons in *L. monocytogenes* have a similar structure, with a DNA-binding transcriptional regulator, encoded by *cadC*, followed by the efflux pump gene, *cadA*. All *cadA* genes in *L. monocytogenes* encode for efflux pumps belonging to ATP-binding cassette (ABC) family (20). The ABC family of efflux pumps relies on ATP hydrolysis to transport cadmium up its concentration gradient and out of the cell (21).

Phenotypic assessment of cadmium resistance spurred the initial characterization of *L. monocytogenes* plasmids (22); however, there are few recent phenotypic studies and little consistency in the assay format, cadmium salts, or cadmium concentrations used. A common method used in the cadmium studies involves spotting cultures on agar plates supplemented with either a single concentration of cadmium or a range of different concentrations and assessing the bacterial growth after incubation (23–25). Another method is similar to the disk diffusion assay commonly used for antibiotic testing, where filters impregnated with cadmium salt solutions are placed onto Petri dishes seeded with a high level of bacteria and a zone of inhibition is measured after incubation (26). Both of these methods provide semi-quantitative results. A more quantitative method is similar to an antibiotic broth dilution assay that evaluates the growth kinetics of bacteria inoculated in liquid media containing a range of cadmium salt concentrations (26). The broth diffusion assay, used in combination with a spectrophotometer, facilitates the collection of data points to create growth curves based on changes in optical density, which can then be modeled to determine lag phase duration (LPD), maximum growth rate, and/or maximum cell density. Measuring these parameters supports quantitative and statistical comparisons of cadmium response, including the impacts of cation concentration on individual strains or differences between strains.

Here, we examined the effect of cadmium salts on the growth behavior of closely (<16 SNPs) and distantly related *L. monocytogenes*. We primarily tested a set of isolates (*n* = 88) from five different dairy facilities collected over the span of a decade, from 2007 to 2017. A majority of the strains within this culture set were isolated from a single facility over a span of 7 years. Prior genomic analysis found that 63 of these strains had very limited genetic variation (<16 SNPs; core genome) and were considered to represent a persistent clonal group (4). Because extensive phenotypic assessment of cadmium resistance is rare in the literature, these data contribute to better understanding of the interplay between cadmium tolerance and the persistence of *L. monocytogenes*.

## MATERIALS AND METHODS

### Bacterial strains and inoculum preparation

*Listeria monocytogenes* isolates previously identified as possessing variants of the *cadAC* operon were used to assess the ability of strains to grow in the presence of different cadmium salts and different concentrations (Table 1). A larger set of *Listeria monocytogenes* strains (*n* = 88; Table S1) previously isolated from dairy processing facilities in British Columbia, Canada, between 2010 and 2017 (4) were used to evaluate strain-level variability in response to cadmium exposure, particularly between strains possessing *cadA1C1*.

**TABLE 1:** *Listeria monocytogenes* strains possessing variants of the *cadAC* operon that were used in this study

*cadAC* variant	Strain	ST^*a*^/CT^*b*^	Isolate source	Isolate reference/accession no.	*cadAC* reference
*cadA1C1*	WRLP46	11/6558	Cheese	R. B. Brown et al. (4)/PRJNA998448	R. B. Brown et al. (4)
*cadA2C2*	WRLP81	288/6544	Environmental	R. B. Brown et al. (4)/PRJNA998448	R. B. Brown et al. (4)
*cadA3C3*	EGDe (ATCC BAA 679)	35/637	Clinical (animal)	G. D. Murray et al. (27)/AL591824	Camejo et al. (15)
*cadA4C4*	Scott A	290/416	Clinical (human)	W. M. D. Fleming et al. (28)/CP023862	Briers et al. (16)
*cadA^-^*	WRLP85	399/1088	Cheese	R. B. Brown et al. (4)/PRJNA998448	R. B. Brown et al. (4)

^*a*^ Sequence type (ST) based on allelic profile of seven housekeeping genes: *acbZ, bglA, cat, dapE, dat, ldh,* and *ihkA* (29).

^*b*^ cgMLST (CT types) are based on allelic differences in 1,748 loci (30).

Isolates were resuscitated from frozen stock culture (−80°C) in tryptic soy broth (TSB, Neogen, Lansing, MI) with incubation at 37°C for 24 h. Cultures were then streaked on tryptic soy agar (TSA, Neogen) and incubated at 37°C for 24 h. A single isolated colony was transferred to TSB and incubated at 37°C for 24 h. The overnight culture was diluted in Mueller-Hinton broth (MHB, BD Difco, Franklin Lakes, NJ) to achieve a cell density of approximately 1 × 10^7^ CFU/mL (colony forming units/mL). This diluted culture served as the inoculum for MIC and growth kinetic studies. Inoculum was used immediately after preparation (within 1 h).

### MIC and kinetic growth parameters

The impact of cadmium on the growth kinetics of *L. monocytogenes* strains was determined using a 96-well plate assay. Cadmium salts, CdCl_2_ (MilliporeSigma, St. Louis, Missouri) and CdSO_4_ (MilliporeSigma, St. Louis, Missouri), were dissolved in MHB to create stock solutions (750 mM) and filter sterilized using a 0.45 mm filter (VWR, Radnor, Pennsylvania). The stock solutions were diluted to targeted test concentrations (40 µM–120 µM) using MHB. MHB + Cd^2+^ solutions were aliquoted (195 µL) into a 96-well plate (Corning Falcon, Corning, NY). MHB without the addition of Cd^2+^ served as the control medium. Wells were inoculated with *L. monocytogenes* inoculum (5 µL) to achieve an approximate initial cell density of 1 × 10^5^ CFU/mL. The 96-well plate was placed in a 96-well spectrophotometer (FilterMax F5, Molecular Devices, San Jose, CA) for controlled incubation at 37°C for 24 h. These conditions are representative of optimum growth conditions for *L. monocytogenes* and were selected to support comparisons with prior studies (18, 23–26, 31–38). Optical densities (OD_595nm_) were recorded every 10 min after a 3-s plate agitation. Growth curve determinations for *cadAC* variants were performed in triplicate for each treatment combination, with experiments repeated on 2 separate days (biological replication). MICs were assigned for each strain as the minimum Cd^2+^ concentration that prevented growth (no significant increase in OD_595nm_ compared to baseline) throughout the 24-h incubation period. *L. monocytogenes* isolates from dairy facilities (*n* = 88) were screened for phenotypic cadmium tolerance based on their ability to grow in the presence of 8 ppm (43.8 µM) CdCl_2_. Screening experiments were performed in triplicate within the same 96-well plate (technical replicates).

The majority of growth curves (93.4%) were as expected; however, aberrant growth patterns were observed in 6.6% of wells and classified into four categories: baseline drift, baseline shift, late control, and erratic early growth. Wells with a baseline drift were deemed irremediable and removed (*n* = 2). Wells with a baseline shift were defined as having growth that was noticeably shifted higher and were corrected by normalizing with another well (*n* = 15). Late control wells were noticed where one control well started growing noticeably later than the other two wells and were ultimately removed (*n* = 2). Erratic early growth was deemed to be associated with a measurement error by the spectrophotometer. When the erratic nature was resolved by 5.8 h (early in lag phase), these early OD values were deleted, and the growth curve reconstructed (*n* = 9). When the erratic nature had not resolved itself by 5.8 h, the well was removed (*n* = 7). Using the adjusted growth curves data (*n* = 517), growth curve parameters (LPD [h], and maximum cell density [OD]) were calculated using the nonlinear fit curve (mechanistic growth model with inverse prediction; OD cutoff = 0.11, inverse prediction OD = 0.09) in JMP Pro 16.0.0 (SAS Institute, Cary, NC). OD values were selected by preliminary visualization of the entire data set to ensure all samples with exponential growth were included in the analysis. Due to variability in starting cell densities between strains and replicates, LPDs of control wells were subtracted from the LPD of MHB + Cd wells for each individual strain. These values were reported as increase in LPD and they were used for all statistical comparisons.

A multiple linear regression model was generated to assess differences between the two tested cadmium salts, CdCl_2_ and CdSO_4_, while also controlling for the effects of different concentrations and cadmium genes. The linear model was used where the cadmium salt serves as an indicator variable.


$$\mu \left(LPD|Concentration,cadGene_{cadA1,cadA2,cadA3,cadA4},CdSalt\right)=\beta_{0}+\beta_{1}Concentration+\beta_{2}cadGene_{cadA1,cadA2,cadA3,cadA4}+\beta_{3}CdSalt$$

Interaction terms were not included as their addition unnecessarily increased the complexity of the model. All statistical analyses and graphs were created using R (4.1.3) and RStudio (2023.06.0+421, Posit, Boston, MA) with the tidyverse (1.3.2) package.

### Long-read sequencing and genome resolution of selected *L. monocytogenes* strains

All the *L. monocytogenes* strains used in this study had previously been whole genome sequenced using short-read sequencing technology by our laboratory or by other investigators (Tables 1 and 2) (4). Six *L. monocytogenes* strains displaying relative differences in cadmium response (slow growth – WRLP95, LPD increase >5 h; intermediate growth – WRLP17, WRLP46, and WRLP77, LPD increase 2–3 h; fast growth – WRLP76 and WRLP81, LPD increase <2 h) were selected for genome resolution using long-read sequencing. Strains were resuscitated from frozen stock in TSB with 0.6% yeast extract (TSBYE; Neogen, Lansing, MI), incubated at 37°C for 24 h. Cultures were then streaked for isolation on TSA with 0.6% yeast extract (Neogen, Lansing, MI), incubated at 37°C for 24 h. A single colony was transferred to TSBYE and incubated at 37°C for 24 h to achieve high cell density. High molecular weight DNA was extracted using the Qiagen Blood & Cell Culture DNA kit (Qiagen, Hilden, Germany) with Qiagen Genomic-tip 100/G following the manufacturer’s protocol. DNA extracts were submitted to Oregon State University’s Center for Quantitative Life Sciences (Corvallis, OR) for genomic DNA quality determination (concentration and size distribution) using the Agilent TapeStation 4200 (Santa Clara, CA). DNA extracts were prepared for long-read sequencing using the Native Barcoding Kit (Oxford Nanopore Technologies, Oxford, UK) with the Rapid Sequencing Barcoding protocol. Sequencing was performed using the MinION flow cell with R10.4.1 chemistry type.

**TABLE 2:** All annotated soft metal resistance genes, including known *cadA* genes with associated protein lengths^*a*^

Cadmium-associated gene(s) with gene label and protein length		WRLP85	WRLP46	WRLP95	WRLP81	EGD-e	Scott A
Chromosome size		2.9 Mb^*b*^	3.0 Mb^*b*^	3.1 Mb^*b*^	3.0 Mb^*b*^	2.9 Mb^*c*^	3.0 Mb^*d*^
Plasmid size		N/A	8.6 Kb^*b*^	6.4 Kb^*e*^	6.9 Kb^*e*^	N/A	N/A
Cadmium efflux ATPase	*cadA1 –* 711 aa	-	P	P	*-*	-	-
	*cadA2 –* 705 aa	-	-	-	P	-	-
	*cadA3 –* 707 aa	-	-	-	-	C	-
	*cadA4 –* 700 aa	-	-	-	-	-	C
Cadmium efflux accessory protein	*cadC1* – 119 aa	-	P	P	*-*	-	-
	*cadC2 –* 119 aa	-	-	-	P	-	-
	*cadC3* – 111 aa	-	-	-	-	C	-
	*cadC4 –* 118 aa	-	-	-	-	-	C
Cadmium resistance protein	133 aa	-	P	-	-	-	-
Pb, Cd, Zn, Hg transporting ATPase location	626 aa	C	C	C	C	C	C
	627 aa	-	P	P	P	-	-
	653 aa	-	-	-	P	-	-
	681 aa	-	-	P	-	-	-
	737 aa	C	C	C	C	C	C
Co, Zn, Cd resistance protein	289 aa	C	C	C	C	C	C
	291 aa	C	C	C	C	C	C
	*czcD –* 303 aa	C	C	C	C	C	C

^*a*^ *L. monocytogenes* strains are ordered by *cadA* gene starting with WRLP85 (*cadA^-^*) up to Scott A (*cadA4*^+^). Strains are noted as having the gene chromosomally (C), on a plasmid (P) or not at all (-).

^*b*^ Length from short-read assembly.

^*c*^ GenBank accession no. AL591824.

^*d*^ GenBank accession no. CP023862.

^*e*^ Length from hybrid assembly.

Genome assembly and annotation were performed using Bacterial and Viral Bioinformatics Resource Center (BV-BRC) Comprehensive Genome Analysis (39) with 50,000 of the raw reads from the long-read sequencing (described above) and previously reported short-read sequencing of these strains (BioProject PRJNA998448) (4). Sequences were assembled using Unicycler v.0.4.8 with the following parameters: trimming reads before assembly; two racon iterations; two pilon iterations; a minimum contig length of 300 bp; and minimum contig coverage of five (40). Unicycler was chosen as it has been shown to increase the likelihood of plasmid recovery (41). Genomes were annotated using RAST tool kit v.1.3.0 (42). Genomes and plasmids were visualized and compared using BLAST Ring Image Generator (BRIG; v.0.95) (11). Multiple sequence alignments were performed using MAFFT v.7.4.5 on BV-BRC (39, 43). Variation analysis on the clonal group was performed using BV-BRC using BWA-mem v.0.7.17 as the aligner and FreeBayes v.1.3.6 for SNP calling (39, 44, 45).

## RESULTS AND DISCUSSION

### Growth behavior of *L. monocytogenes* strains with unique *cadAC* variants and their response to different cadmium salts

**Fig 1:**
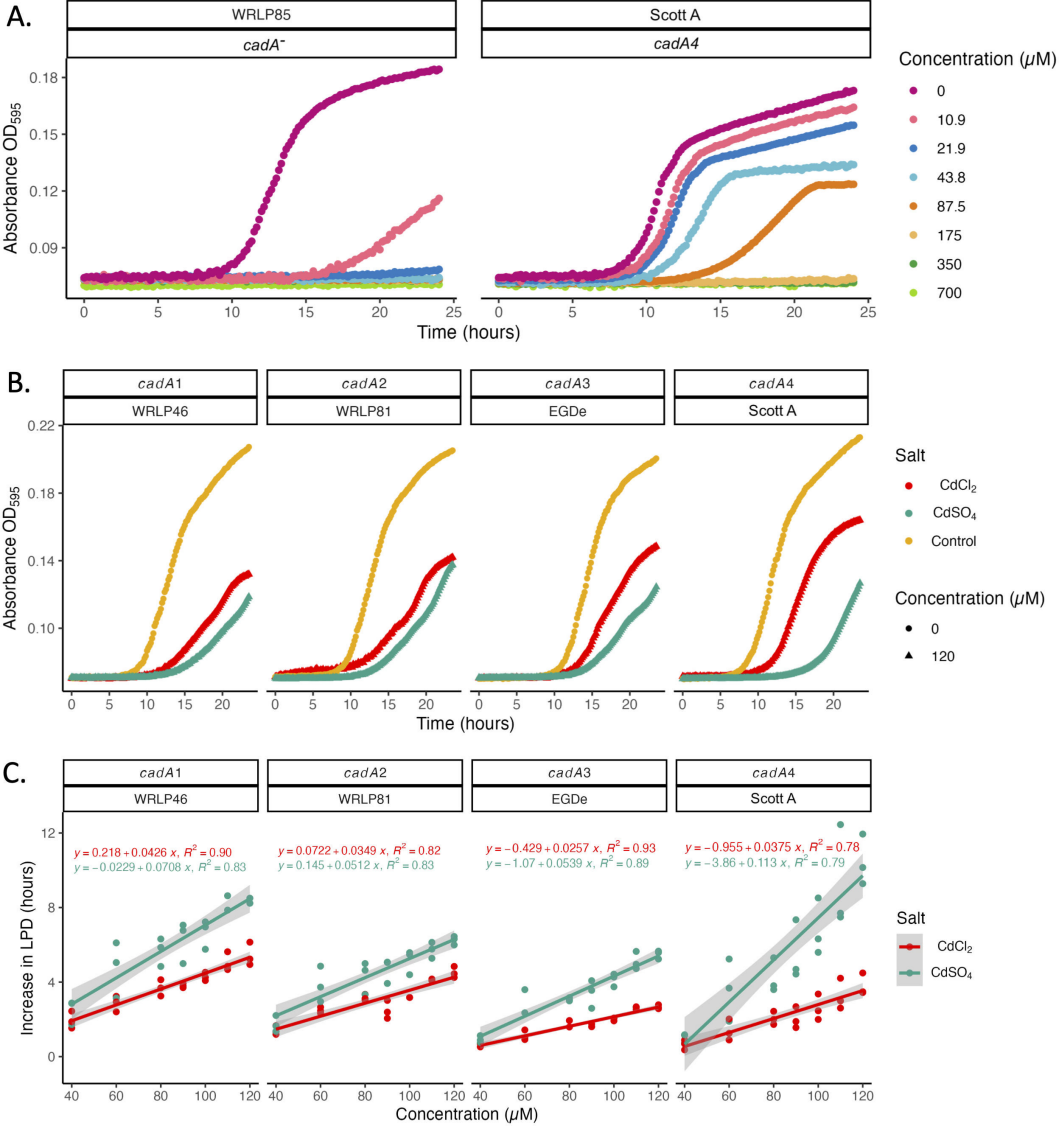
(**A**) Growth of *Listeria monocytogenes* WRLP85 (*cadA*^-^) and Scott A (*cadA4*^+^) in the presence of 0 µM–700 µM CdCl_2_. Initial inoculum was 5 log CFU/mL in MHB, incubated at 37°C for 24 h. Growth curves represent the average of three replicates at each dose. (**B**) Effect of different cadmium salts on the growth of *L. monocytogenes* strains, each with a unique *cadA* resistance gene. Growth curves are an average of three replicates grown in MHB (control) or in MHB supplemented with 120 µM of CdCl_2_ or CdSO_4_. (**C**) Linear regression model of the increase in LPD of *L. monocytogenes* strains with unique *cadA* variants in response to growth in the presence of CdCl_2_ or CdSO_4_ at concentrations ranging between 40 and 120 µM.

All four *cadA* (*cadA1–cadA4*) variants tested managed CdCl_2_ more effectively than CdSO_4_. This was unexpected as the two salts dissolve readily in water although their overall solubility is different, at 133 mg/mL and 75 mg/mL, respectively (Fig. 1).

Representative CdCl_2_ dose response curves for the growth of the *cadA^-^* strains (WRLP85) compared to the *cadA4*^+^ Scott A strain are shown in Fig. 1A. The *cadA^-^* isolate, WRLP85, was able to tolerate a small amount of CdCl_2_ (MIC 21.9 µM), whereas Scott A (*cadA4*) possessed highest tolerance, at MIC of 175 µM. WRLP10, possessing *cadA1*, had a CdCl_2_ MIC of 95.4 µM. Similar levels of cadmium tolerance in *L. monocytogenes*, when tested in broth, were reported in the literature (46). In contrast, the MIC values seen in the present study were far below the values reported in the literature when using agar-based methods. Previous studies have used 382 µM (70 ppm) CdCl_2_ as the MIC cutoff determination, where isolates that were able to grow at or above this concentration were deemed tolerant to cadmium (18, 31, 33, 34). In the original literature, these isolates were called “resistant”; however, considering the concentrations tested and controversy regarding concentrations that would deem isolates resistant (47) for the consistency purpose here, we refer to them as “tolerant.” This is in agreement with L. Jiang (48), who reported that microbroth dilution assays generally yield lower MIC results compared to agar dilution methods. It may be that the diffusion of cadmium in broth is higher than on agar due to Brownian motion, which also increases the chances of impact with the bacterial membrane as well as its fluidity.

The ability of the *cadA^-^* isolate WRLP85 to grow in 10.9 µM of cadmium implies a baseline tolerance of *L. monocytogenes* to cadmium. A previous study had similar findings; when *cadA* was deleted, the MIC decreased from 100 µM in the wild-type strain to 10 µM in the mutant strain (26).

Increasing the CdCl_2_ dose caused corresponding increases in LPD, with growth observed at concentrations up to 87.5 µM for Scott A (Fig. 1A). For the same strain, no growth was seen at 175 µM CdCl_2_ (MIC), indicating that this concentration of cadmium overwhelmed the ability of cadmium tolerance mechanisms to overcome this stress. At concentrations of at least 43.8 µM, Scott A also exhibited a decrease in the final OD, suggesting potential ATP expenditure to support efflux pump activity at the expense of biomass production. Similar observations have been made in *Pseudomonas aeruginosa* during antibiotic resistance development, where the over-expression of an efflux pump is associated with the increased expression of the nitrate respiratory chain to make up for the increased energy demand (49).

The most common cadmium salts used in the studies are CdCl_2_ and CdSO_4_; however, there has been little investigation into the effect of two compounds on *L. monocytogenes* phenotype. When we exposed *L. monocytogenes* strains possessing different *cadA* variants to CdCl_2_ and CdSO_4_, at concentrations between 40 and 120 µM, there was a significant difference in LPD of strains at 120 µM for the tested cadmium salts (Fig. 1B). Notably, the effect was the most pronounced for the *cadA4^+^* strain (Scott A; the strain with the highest CdCl_2_ MIC) with an average LPD increase of 2.26 h for CdCl_2_ compared to 5.83 h for CdSO_4_.

The individual regression graphs demonstrated that Scott A (*cadA4*^+^) struggled to adapt to increasingly higher concentrations of CdSO_4_ when compared to the strains carrying the other cadmium resistance genes (Fig. 1C). WRLP81, which possesses *cadA2,* had the smallest differences in LPD between the two cadmium salts as shown by the slope of the regression lines (CdCl_2_: 0.03 h/µM vs CdSO_4_: 0.05 h/µM). Using a multiple linear regression model, on average CdSO_4_ increased strains’ LPD over CdCl_2_ by 2.25 h (± 0.34, 95% CI), when controlling for the effect of the different cadmium genes and holding the concentration constant (*P* ≤ 0.05). A more complex model considering the various slopes of the different cadmium genes incurred a loss of precision and interpretability due to redundant terms. Caution should be used if applying this model for future predictions, especially for isolates such as Scott A, that are particularly sensitive to CdSO_4_ concentration.

Prior studies on cadmium tolerance most commonly used CdCl_2_ or CdSO_4_; however, the two salts have only been compared against *L. monocytogenes* by R. Pombinho et al. (26). Using a disk diffusion assay, they found no difference in response between the two salts; however, this method is unlikely to provide the sensitivity to discern differences. Since cadmium salts impact the growth kinetics of *L. monocytogenes,* methods that evaluate growth at an endpoint, such as disk diffusion assays and agar dilutions, would be minimally influenced by cadmium salt selection, assuming incubation time was sufficient. Cadmium salt hydrates, including CdSO_4_•8H_2_O and CdCl_2_•H_2_O, have also been used in prior studies, albeit far less commonly (22, 25). Most cadmium studies use a single salt and report concentration as a function of mass (ppm), making conversion to molarity essential prior to comparing data across studies.

### Screening *L. monocytogenes* isolates recovered from Canadian dairy facilities from 2007 to 2017 for cadmium tolerance

**Fig 2:**
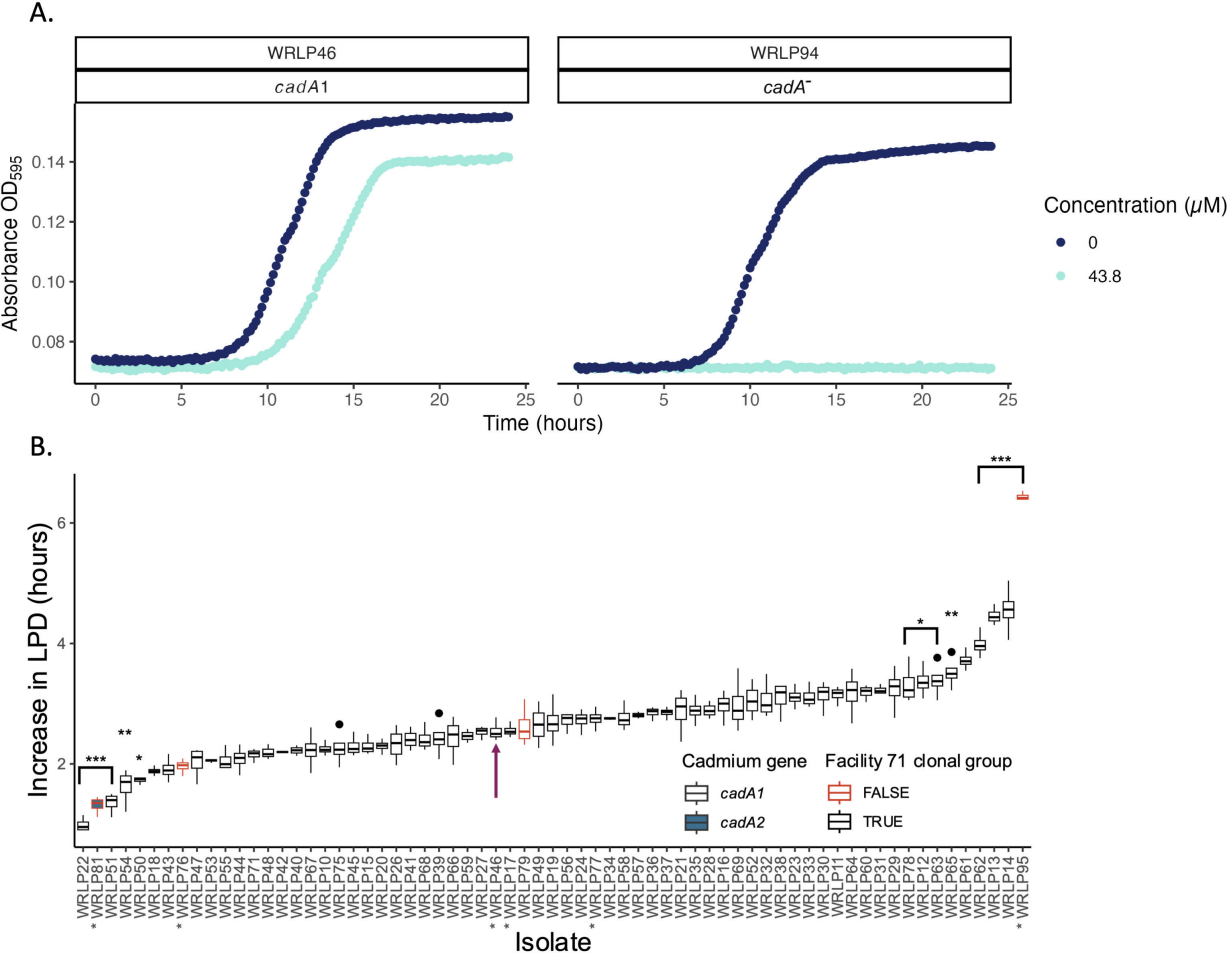
(**A**) Representative growth curves of *cadA*^+^ and *cadA*^-^ *L. monocytogenes* strains isolated from dairy processing facilities in B.C., Canada, between 2007 and 2017 in the presence of 0 or 43.8 µM CdCl_2_. Inoculum was approximately 5 log CFU/mL in MHB, incubated at 37°C for 24 h. Growth curves represent the average of three replicates. (**B**) Increase in LPD for *L. monocytogenes* isolates with *cadA*. Data for strains that are part of the clonal group originally isolated from facility 71 are shown in black, whereas data for isolates outside of this clonal group are shown in red. Fill color indicates which *cadA* variant each possesses; white has *cadA1,* while teal represents *cadA2*. Isolates that were selected for long-read sequencing and genome resolution are identified with an asterisk underneath to the isolate ID. Data represent LPD from three independent replicates comparing growth in the presence of 0 and 43.8 µM CdCl_2_. The asterisks above the associated isolates represent groupings from a Dunnett *post hoc* test using WRLP46, indicated with a maroon arrow, as the reference. The number of asterisks is associated with levels of significance; *P* < 0.001 (***), *P* < 0.01 (**), *P* < 0.05 (*).

Based on cadmium growth kinetics for control strains with various *cadA* variants, 43.8 µM (8 ppm) of CdCl_2_ was selected as the concentration for screening *L. monocytogenes* isolates from dairy processing facilities (*n* = 88) for cadmium tolerance. Representative growth curves for *cadA1^+^* (WRLP 46) and *cadA*^-^ (WRLP 94) strains are shown in Fig. 2A. At this concentration, none of the *cadA^-^* isolates were able to grow while all *cadA^+^* (*n* = 67) isolates grew, albeit with a significant delay compared to MHB without added cadmium (*P* < 0.05, two sample *t*-test). As *cadA^-^* isolates failed to grow, they were omitted from subsequent data analysis. The average LPD for control wells was 8.54 ± 0.79 h compared to 11.26 ± 1.08 h average LPD for *cadA*^+^ isolates grown in the presence of cadmium. *cadA^+^* strains also exhibited a significantly lower final OD when grown in the presence of 43.8 µM CdCl_2_ compared to no cadmium (*P* < 0.05, two sample *t*-test). The average final OD reading for strains grown in the presence of cadmium was 0.128 ± 0.03 compared to 0.172 ± 0.01 for no cadmium controls.

**Fig 3:**
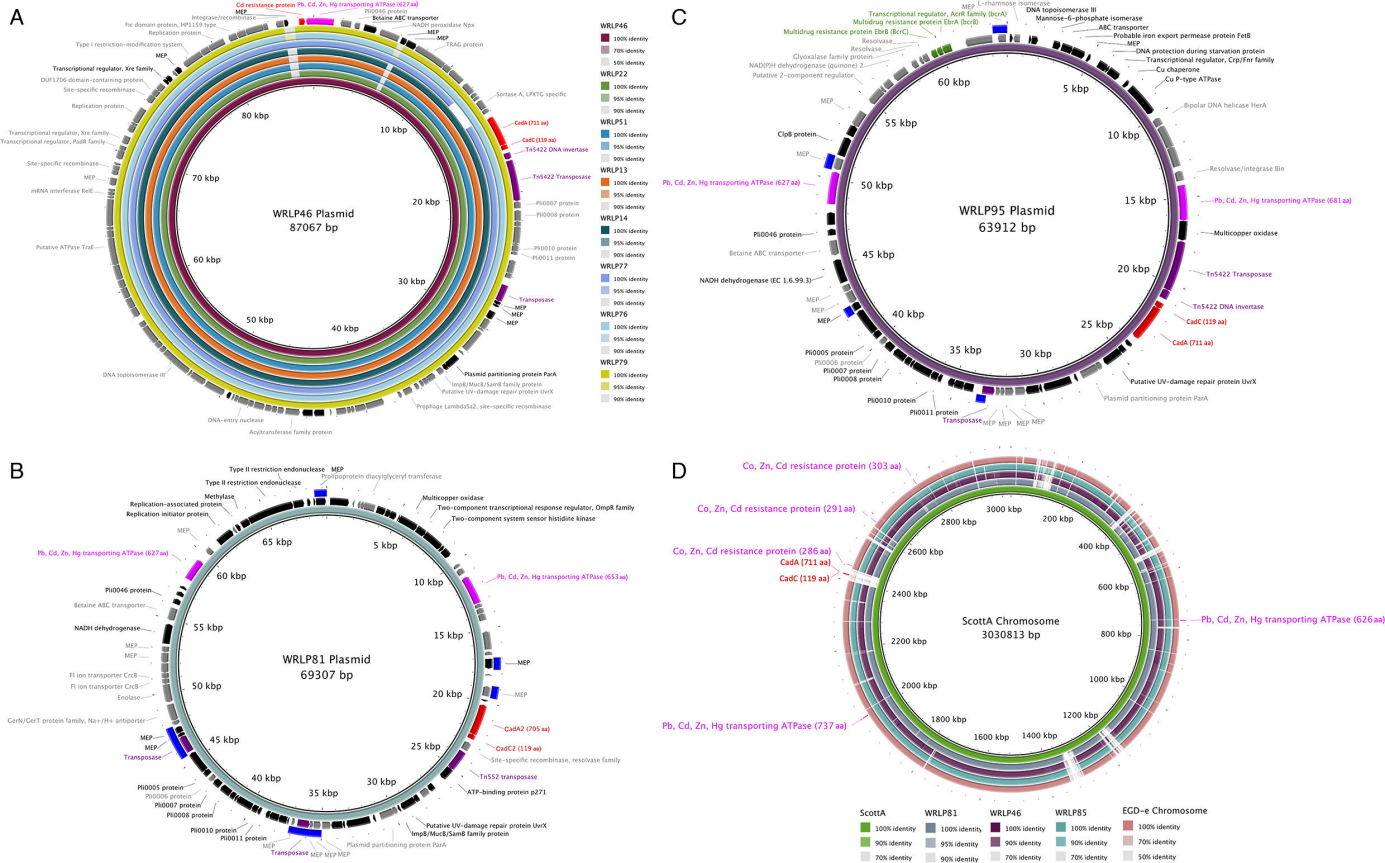
(**A**) BRIG map of plasmids shared by isolates WRLP46 (reference genome), WRLP22, WRLP51, WRLP13, WRLP14, WRLP77, WRLP76, and WRLP79. Plasmid maps were constructed using hybrid assembly with long- and short-read sequencing data. Black arrows denoted on the outer ring represent the positive DNA strand while gray is the negative strand. MEP are mobile element proteins. Unlabeled arrows are hypothetical proteins. Cadmium-specific genes are highlighted in red. Non-specific soft metal-associated annotations are highlighted in fuchsia, and purple are highlighted transposases. (**B**) BRIG map of the plasmid of WRLP81 carrying *cadA2*. Blue squares are repeat regions. (**C**) BRIG map of the plasmid of WRLP95 carrying *cadA1* with *bcrABC* highlighted in green. (**D**) BRIG map using the chromosome of Scott A (*cadA4*; GenBank accession no. CP023862) as a reference to map the chromosomes of WRLP46 (*cadA1*), WRLP81 (*cadA2*), EGDe (*cadA3*; GenBank accession no. AL591824), and WRLP85 (*cadA^-^*).

Increase in LPD due to the presence of 43.8 µM of CdCl_2_ of *cadA*^+^ dairy isolates is shown in Fig. 2B. Within the tested isolate set, 63/67 *cadA*^+^ isolates were part of a persistent clonal group (<16 SNPs, core genome) (4), isolated from a single facility. Two additional strains (WRLP76, WRLP79) were isolated from the same facility, years apart, and very closely related (<33 SNPs from clonal group). All of these strains carried *cadA1C1* on an 87 kb plasmid (Fig. 3A). They also carried a cadmium resistance protein (133 aa; Fig. 3A) adjacent to the 627 aa soft metal ATPase on the same plasmid. Compared to other *Listeria* sequences in the NCBI database using BLASTp, the cadmium resistance protein found in these isolates is truncated, with the first 53 aa missing.

The average LPD increase in the presence of cadmium for strains in this persistent cluster was 2.69 ± 0.7 h. For statistical analysis purposes, WRLP46 was selected as a strain representing an average increase in LPD (2.55 ± 0.2 h) when grown in the presence of CdCl_2_. The LDP of WRLP22 (0.99 ± 0.14 h) and WRLP51 (1.35 ± 0.2 h) was significantly less impacted by cadmium when compared to WRLP46 (*P* < 0.001). The presence of cadmium led to significantly higher LPD for WRLP62 (3.99 ± 0.26 h), WRLP13 (4.45 ± 0.18 h), and WRLP14 (4.56 ± 0.49 h) compared to WRLP46.

Plasmid mapping and variation analysis of these isolates demonstrated that the plasmids have identical sequences (0 SNPs; Fig. 3A). Further evaluation of the plasmids in the entire persistent clonal group revealed that WRLP77 was the only isolate that possessed a plasmid with a variation in the sequence; a ~2,100 bp deletion was seen immediately downstream of the Tn*5422* cluster containing *cadA1*.

SNP variation analysis between WRLP46 and isolates that exhibited either highest (WRLP13, WRLP14) or lowest LPD increase (WRLP22, WRLP51) in the presence of cadmium was also performed. WRLP13 and WRLP14 had two conserved non-synonymous SNPs in an NtrC family transcriptional regulator (I746T) and a sodium/hydrogen exchanger family protein (E54D). Two of the fastest growing isolates that belong to the clonal group, WRLP22 and WRLP51, only had conserved synonymous SNPs in phage components. There were additional SNPs identified (WRLP51, 18 SNPs; WRLP14, 46 SNPs); however, no patterns based on LPD differences were observed (Fig. 2B; Table S2). Similarly, the comparative systems analysis identifying the presence/absence of genes within this strain set provided limited information. Only five coding regions differed across the set, with two being associated with phage structural components, one putative integrase, and two hypothetical proteins. There was no pattern based on LPD differences.

The two remaining *cadA*^+^ dairy isolates, WRLP81 and WRLP95, were isolated from other dairy facilities and possessed *cadA1* and *cadA2* on 69 kb and 64 kb plasmids, respectively (Fig. 3B and C). The growth of WRLP95 was particularly impacted by cadmium, with an increase in LPD of 6.44 ± 0.08 h (Table S2) despite having identical *cadA1* sequences to all other strains in the set. This discrepancy among *cadA*^+^ isolates has been seen before, with up to a 860 µg/mL (4691 µM, converted) difference in MICs between two *cadA1* isolates (24). Additionally, variation in gene expression has been seen in closely related strains of the same serotype (50). The *cadA1* gene in WRLP95 was conserved in association with Tn*5422* but in an opposite orientation compared to WRLP46 (Fig. 3C). These plasmids share limited homology (33%). This is predominantly in the regions associated with Tn*5422* as well as the region containing the 627 aa soft metal ATPase, Pli0046, betaine ABC transporter, and the NADH dehydrogenase, likely components of a mobile element. WRLP95 was the only isolate within the set to also carry a *bcrABC* cassette, associated with an efflux pump that confers tolerance to quaternary ammonium compounds (QACs). Research has shown a correlation between *cadA* genes and the QAC tolerance gene *bcrABC*, and a potential link between these genes and increased likelihood of isolate persistence in food production environments (3, 5). In *L. monocytogenes* isolates with *bcrABC*, it is highly likely the isolates will also possess a *cadA* gene; however, the reverse is not as consistent (31, 34, 51). This is in line with our data, where isolates within the same clonal group all possessed *cadA1*, but only one had *bcrABC*. The co-selection of these two genes was also demonstrated previously, when the transfer of both *bcrABC* and *cadA* to *L. monocytogenes* was consistent enough that acquiring *cadA* was an indicator that an isolate had also received *bcrABC* (38).

WRLP81, the only *cadA2*^+^ isolate in the strain set, showed high tolerance to cadmium with LPD of 1.32 ± 0.17 h. It was grouped with more cadmium-tolerant *cadA1* isolates. This is not surprising, considering *cadA1* and *cadA2* share 70% aa identity (Fig. S1) and have been shown to have similar MICs (18, 46).

### Genomic properties of control and *cadA*^-^ strains

The chromosomes of the control strains (WRLP85, WRLP46, WRLP81, and EGD-e) were mapped to *L. monocytogenes* Scott A (*cadA4*^+^), and genes associated with soft metal tolerance are shown in Fig. 3D. *L. monocytogenes* EGD-e, Scott A, and WRLP85 do not carry plasmids. Plasmid maps for WRLP81 (*cadA2*^+^) and WRLP95 (*cadA1*+) are displayed in Fig. 3B and C, respectively. The plasmid map of WRLP46 (*cadA1*^+^) and additional representative isolates from clonal group isolated from Facility 71 (e.g., increased cadmium tolerance: WRLP22, WRLP51; reduced cadmium tolerance: WRLP13, WRLP14) as well as two closely related isolates from Facility 71 (WRLP76 and WRLP79) are shown in Fig. 3A. Additional details related to soft metal-associated genes for the control strains are highlighted on the chromosome and plasmid maps and presented in Table 2. Other genetic features of interest (i.e., transposases, repeat regions, and the sanitizer efflux pump *bcrABC*) are also highlighted on the plasmid maps.

The most well-studied of the soft metal-associated genes in *L. monocytogenes* are the cadmium-specific efflux pumps (*cadA*) and their nearby regulators (*cadC*). These cassettes share reasonably similar homology (Fig. S1), but they differ in their mobility and localization within the genome. Scott A possesses the *cadA4C4* variant located on the *Listeria* genomic island 2, which also carries several genes associated with arsenic tolerance in the 2.41 Mb region (16). EGD-e carries the *cadA3, ispB,* and *cadC3* cassette within an integrative and conjugative element in a distant location on the chromosome (~1.13 Mb) (15). WRLP81 carries *cadA2C2* on a putative Tn*552* within a ~69 kb plasmid (Fig. 3B). WRLP95 possesses *cadA1C1* on a ~64 kb plasmid (Fig. 3D). WRLP46 and 65 of the other dairy isolates from Facility 71 also carry *cadA1C1*, located on a ~87 kb plasmid (Fig. 3A). Both plasmids carrying *cadA1C1* are associated with Tn*5422*; however, the two plasmids share only 33% homology. Many of the *L. monocytogenes* strains, including WRLP85 and 21 of the other dairy isolates, do not harbor any plasmids. These strains do not possess a *cadAC* cassette within their genomes.

All six examined *L. monocytogenes* strains carry three small cobalt, zinc, and cadmium resistance proteins (286, 291, and 303 aa) on the chromosome, encoded by *czc* genes. *L. monocytogenes* serotype 1/2a isolates (e.g., EGD-e, WRLP46, WRLP85, and WRLP95) have identical predicted amino acid sequences for all three *czc* genes, whereas sequences for Scott A (serotype 4b) and WRLP81 (serotype 1/2b) differ from the serotype 1/2a isolates but are identical to one another. Predicted amino acid sequences for the *czc* genes differ between the two groups by 4 (Q138R, F163L, R242K, E262A), 1 (T222A), and 1 (S256T) amino acid for the 286, 291, and 303 aa proteins, respectively.

These *czc* genes are dispersed throughout the chromosome but are highly conserved within their own groups with the *czc* genes associated with 286 aa and 291 aa proteins existing within conserved regions of >45,000 bp and >25,000 bp, respectively. The *czc* gene (*czcD*) with 303 aa protein length also sits in a highly conserved region of >30,000 bp; however, there are two options for genes immediately upstream of the *czcD* and downstream of a Cof-like hydrolase: (i) a putative peptidoglycan bound protein (LPTXG motif) (EGD-e, WRLP46, WRLP85, WRLP95) or (ii) a mobile element protein (Scott A, WRLP81). *czcD* has been shown to have a significant increase in expression directly after cadmium exposure that soon levels off (52). It was hypothesized that *czcD* was therefore a regulatory gene that was upregulated upon exposure to activate the relevant genes (52). Given the size of CzcD, it is unlikely that it is an efflux pump; however, it is somewhat similar in size to the CadA regulatory protein, CadC (protein length 119 aa for CadC1). Due to the high conservation of *czc* genes, these genes likely play a role in low-level cadmium tolerance of *L. monocytogenes*.

All six strains also carry two soft metal efflux ATPases on the chromosome (626 aa and 737 aa long). The 626 aa protein is in a highly conserved region (20,000 bp) and the 737 aa protein is in a conserved region of >65,000 bp. Both have the conserved DKTGTLT ATP phosphorylation site for P-type ATPases; however, only the 737 aa protein has the CXXC metal binding motif (20, 53, 54). Considering the longer 737 aa ATPase has both requisite ATP phosphorylation site and the heavy metal binding site, it is a possible reason for the inherent cadmium tolerance observed in the *cadA^-^* isolate, WRLP85. The shorter of the two proteins, 626 aa, lacks the CXXC metal binding motif, making it less likely to contribute to cadmium tolerance (54). To our knowledge, no one has tested either of these efflux pumps phenotypically in *L. monocytogenes*. Again, homology of these proteins was conserved within the serogrouping.

WRLP46 plasmid, and by extension of the rest of the plasmid-harboring isolates from Facility 71, carried a (third) soft metal efflux ATPase (627 aa) adjacent to a short protein (133 aa) that was annotated as a “cadmium resistance protein.” This short “cadmium resistance protein” shares a low homology (24.3% aa identity) with CadX, a negative transcriptional regulator in *Streptococcus salivarius* (55). WRLP95 and WRLP81 plasmids also carry the 627 aa soft metal ATPase and both plasmids carry a second (fourth), but different, soft metal ATPase (WRLP95, 681 aa; WRLP81, 653 aa). Alignments of these soft metal ATPases are provided in Fig. S1. None of the three of proteins (627 aa, 681 aa, 653 aa) have the metal binding motif and only the 627 aa and 681 aa proteins have the ATP phosphorylation site. It is unclear if any of these other annotated ATPases play a role in conferring cadmium tolerance in isolates that possess them; however, the only metal ATPase in isolates WRLP95 and WRLP81 other than *cadA* to have both the necessary CXXC metal binding motif and the ATP phosphorylation cite is the 737 aa protein that is shared among all of the isolates listed in Table 2.

### Conclusion

Phenotypic responses of the *L. monocytogenes* isolates examined in the present study varied in the presence of cadmium. When isolates were exposed to two commonly studied cadmium salts, CdCl_2_ and CdSO_4_, the companion anion significantly affected growth kinetics of *L. monocytogenes* strains. Isolates possessing different cadmium resistance genes, namely *cadA1, cadA2, cadA3,* or *cadA4*, were able to grow faster in CdCl_2_ than CdSO_4_. A low level of inherent cadmium tolerance (~10 µM) was also observed in *L. monocytogenes* isolates that do not carry any plasmids associated with *cadA* and other soft metal resistance genes. Despite genetic similarity, within a clonal group with over 60 strains possessing identical plasmids with *cadA* genes, there were phenotypic differences observed in the presence of cadmium. These findings enhance our understanding of *L. monocytogenes* cadmium tolerance; however, further research is needed to discover the underlying genetic and physiological factors involved in cadmium tolerance. To the best of our knowledge, no studies have examined the difference in genetic expression among isolates with small SNP differences. Further transcriptomics testing would be useful to establish differences in gene expression among closely related isolates in relation to their tolerance of cadmium and other compounds encountered in the food production chain.

## Data Availability

The data set presented in this study can be found in the NCBI Sequence Read Archive (SRA) under BioProject PRJNA998448.

## SUPPLEMENTAL MATERIAL

Fig. S1 aem.01281-24-s0001.pdf MAFFT alignments of CadA, CadC, and other annotated cadmium resistance proteins.

Tables S1 and S2 aem.01281-24-s0002.docx Background information on L. monocytogenes isolates used in the study and lag phase duration for cadA+ isolates when exposed to cadmium at 8 ppm.

ASM does not own the copyrights to Supplemental Material that may be linked to, or accessed through, an article. The authors have granted ASM a non-exclusive, world-wide license to publish the Supplemental Material files. Please contact the corresponding author directly for reuse.
