# Supplementary material for: Variability in cadmium tolerance of closely related Listeria monocytogenes isolates originating from dairy processing environments
Source: Appl Environ Microbiol. 2024 Nov 21;91(1):e01281-24. doi: 10.1128/aem.01281-24 (PMC11784300; doi:10.1128/aem.01281-24)
Supplement: Fig. S1 — MAFFT alignments of CadA, CadC, and other annotated cadmium resistance proteins. [file aem.01281-24-s0001.pdf]

Figure S1 | MAFFT alignments of CadA, CadC, and other annotated cadmium resistance proteins.

Reference Sequence: 289aa_WRLP46

```
289aa_WRLP46     MDGYNDLKKAEKAAFLSIFAYLFLAVLKIVAGQLGNSDALLADGLNNTTDIVASVALLIG
289aa_WRLP85     MDGYNDLKKAEKAAFLSIFAYLFLAVLKIVAGQLGNSDALLADGLNNTTDIVASVALLIG
289aa_WRLP95     MDGYNDLKKAEKAAFLSIFAYLFLAVLKIVAGQLGNSDALLADGLNNTTDIVASVALLIG
289aa_EGDe       MDGYNDLKKAEKAAFLSIFAYLFLAVLKIVAGQLGNSDALLADGLNNTTDIVASVALLIG
289aa_WRLP81     MDGYNDLKKAEKAAFLSIFAYLFLAVLKIVAGQLGNSDALLADGLNNTTDIVASVALLIG
289aa_ScottA     MDGYNDLKKAEKAAFLSIFAYLFLAVLKIVAGQLGNSDALLADGLNNTTDIVASVALLIG
                 ************************************************************

289aa_WRLP46     LRISRIPPDADHSYGHRRTETISSLIASIIMFLVGVQVIWSSIVHIIEKEFATPSMLTAV
289aa_WRLP85     LRISRIPPDADHSYGHRRTETISSLIASIIMFLVGVQVIWSSIVHIIEKEFATPSMLTAV
289aa_WRLP95     LRISRIPPDADHSYGHRRTETISSLIASIIMFLVGVQVIWSSIVHIIEKEFATPSMLTAV
289aa_EGDe       LRISRIPPDADHSYGHRRTETISSLIASIIMFLVGVQVIWSSIVHIIEKEFATPSMLTAV
289aa_WRLP81     LRISRIPPDADHSYGHRRTETISSLIASIIMFLVGVQVIWSSIVHIIEKEFATPSMLTAV
289aa_ScottA     LRISRIPPDADHSYGHRRTETISSLIASIIMFLVGVQVIWSSIVHIIEKEFATPSMLTAV
                 ************************************************************

289aa_WRLP46     VALFSGVFMYAIYLYNHQLAKKLDSQAVRAAAYDNRSDAFVSFGAFIGIIGAVLGVPWLD
289aa_WRLP85     VALFSGVFMYAIYLYNHQLAKKLDSQAVRAAAYDNRSDAFVSFGAFIGIIGAVLGVPWLD
289aa_WRLP95     VALFSGVFMYAIYLYNHQLAKKLDSQAVRAAAYDNRSDAFVSFGAFIGIIGAVLGVPWLD
289aa_EGDe       VALFSGVFMYAIYLYNHQLAKKLDSQAVRAAAYDNRSDAFVSFGAFIGIIGAVLGVPWLD
289aa_WRLP81     VALFSGVFMYAIYLYNHRLAKKLDSQAVRAAAYDNRSDAFVSLGAFIGIIGAVLGVPWLD
289aa_ScottA     VALFSGVFMYAIYLYNHRLAKKLDSQAVRAAAYDNRSDAFVSLGAFIGIIGAVLGVPWLD
                 *****************:************************:*****************

289aa_WRLP46     SVTAFLVGILIVYTAIKIFYDAAHTLTDGFDVSKLETIHDLIASVPDVKKVIDIKARMNG
289aa_WRLP85     SVTAFLVGILIVYTAIKIFYDAAHTLTDGFDVSKLETIHDLIASVPDVKKVIDIKARMNG
289aa_WRLP95     SVTAFLVGILIVYTAIKIFYDAAHTLTDGFDVSKLETIHDLIASVPDVKKVIDIKARMNG
289aa_EGDe       SVTAFLVGILIVYTAIKIFYDAAHTLTDGFDVSKLETIHDLIASVPDVKKVIDIKARMNG
289aa_WRLP81     SVTAFLVGILIVYTAIKIFYDAAHTLTDGFDVSKLETIHDLIASVPDVKKVIDIKARMNG
289aa_ScottA     SVTAFLVGILIVYTAIKIFYDAAHTLTDGFDVSKLETIHDLIASVPDVKKVIDIKARMNG
                 ************************************************************

289aa_WRLP46     NRIWIDATIAVDPELNVVKSHEITEIVEQKIRNEYEGAFTLVHIEPFFE
289aa_WRLP85     NRIWIDATIAVDPELNVVKSHEITEIVEQKIRNEYEGAFTLVHIEPFFE
289aa_WRLP95     NRIWIDATIAVDPELNVVKSHEITEIVEQKIRNEYEGAFTLVHIEPFFE
289aa_EGDe       NRIWIDATIAVDPELNVVKSHEITEIVEQKIRNEYEGAFTLVHIEPFFE
289aa_WRLP81     NKIWIDATIAVDPELNVVKSHAITEIVEQKIRNEYEGAFTLVHIEPFFE
289aa_ScottA     NKIWIDATIAVDPELNVVKSHAITEIVEQKIRNEYEGAFTLVHIEPFFE
                 *:******************* ***************************
```

```
Percent Identity  Matrix
     1: 289aa_WRLP46  100.00  100.00  100.00  100.00   98.62   98.62
     2: 289aa_WRLP85  100.00  100.00  100.00  100.00   98.62   98.62
     3: 289aa_WRLP95  100.00  100.00  100.00  100.00   98.62   98.62
     4: 289aa_EGDe    100.00  100.00  100.00  100.00   98.62   98.62
     5: 289aa_WRLP81   98.62   98.62   98.62   98.62  100.00  100.00
     6: 289aa_ScottA   98.62   98.62   98.62   98.62  100.00  100.00
```

Reference Sequence: 291aa_WRLP46

```
291aa_WRLP46    MNHSNLTILSVCSNFVIVVLKLIVGFFTGSVAVISEGIHSSMDLFASIITFFSIRISNQP
291aa_WRLP95    MNHSNLTILSVCSNFVIVVLKLIVGFFTGSVAVISEGIHSSMDLFASIITFFSIRISNQP
291aa_WRLP85    MNHSNLTILSVCSNFVIVVLKLIVGFFTGSVAVISEGIHSSMDLFASIITFFSIRISNQP
291aa_EGDe      MNHSNLTILSVCSNFVIVVLKLIVGFFTGSVAVISEGIHSSMDLFASIITFFSIRISNQP
291aa_WRLP81    MNHSNLTILSVCSNFVIVVLKLIVGFFTGSVAVISEGIHSSMDLFASIITFFSIRISNQP
291aa_ScottA    MNHSNLTILSVCSNFVIVVLKLIVGFFTGSVAVISEGIHSSMDLFASIITFFSIRISNQP
                ************************************************************

291aa_WRLP46    ADEDHPYGHGKAENIAGTIETLLIFVAGIWIIVESVNKLVNPHEIRFPALGIMVMLFGAI
291aa_WRLP95    ADEDHPYGHGKAENIAGTIETLLIFVAGIWIIVESVNKLVNPHEIRFPALGIMVMLFGAI
291aa_WRLP85    ADEDHPYGHGKAENIAGTIETLLIFVAGIWIIVESVNKLVNPHEIRFPALGIMVMLFGAI
291aa_EGDe      ADEDHPYGHGKAENIAGTIETLLIFVAGIWIIVESVNKLVNPHEIRFPALGIMVMLFGAI
291aa_WRLP81    ADEDHPYGHGKAENIAGTIETLLIFVAGIWIIVESVNKLVNPHEIRFPALGIMVMLFGAI
291aa_ScottA    ADEDHPYGHGKAENIAGTIETLLIFVAGIWIIVESVNKLVNPHEIRFPALGIMVMLFGAI
                ************************************************************

291aa_WRLP46    VNIIVSRIIKKAADEANSVAMKSNALHLYTDVFTSLGIALSLFLVYITGWLWLDPVIAIL
291aa_WRLP95    VNIIVSRIIKKAADEANSVAMKSNALHLYTDVFTSLGIALSLFLVYITGWLWLDPVIAIL
291aa_WRLP85    VNIIVSRIIKKAADEANSVAMKSNALHLYTDVFTSLGIALSLFLVYITGWLWLDPVIAIL
291aa_EGDe      VNIIVSRIIKKAADEANSVAMKSNALHLYTDVFTSLGIALSLFLVYITGWLWLDPVIAIL
291aa_WRLP81    VNIIVSRIIKKAADEANSVAMKSNALHLYTDVFTSLGIALSLFLVYITGWLWLDPVIAIL
291aa_ScottA    VNIIVSRIIKKAADEANSVAMKSNALHLYTDVFTSLGIALSLFLVYITGWLWLDPVIAIL
                ************************************************************

291aa_WRLP46    TAFYIMYEAYKLLKESFPPLMDKRLSADEEEAIKQIILAHKTRFIEFHDFRSRRAGAEEY
291aa_WRLP95    TAFYIMYEAYKLLKESFPPLMDKRLSADEEEAIKQIILAHKTRFIEFHDFRSRRAGAEEY
291aa_WRLP85    TAFYIMYEAYKLLKESFPPLMDKRLSADEEEAIKQIILAHKTRFIEFHDFRSRRAGAEEY
291aa_EGDe      TAFYIMYEAYKLLKESFPPLMDKRLSADEEEAIKQIILAHKTRFIEFHDFRSRRAGAEEY
291aa_WRLP81    TAFYIMYEAYKLLKESFPPLMDKRLSADEEEAIKQIILAHKARFIEFHDFRSRRAGAEEY
291aa_ScottA    TAFYIMYEAYKLLKESFPPLMDKRLSADEEEAIKQIILAHKARFIEFHDFRSRRAGAEEY
                *****************************************:******************

291aa_WRLP46    IDFHLVVSSSMTIESAHSLCDEIEAEIMNFYAKAEVLIHLEPEEERVLTRI
291aa_WRLP95    IDFHLVVSSSMTIESAHSLCDEIEAEIMNFYAKAEVLIHLEPEEERVLTRI
291aa_WRLP85    IDFHLVVSSSMTIESAHSLCDEIEAEIMNFYAKAEVLIHLEPEEERVLTRI
291aa_EGDe      IDFHLVVSSSMTIESAHSLCDEIEAEIMNFYAKAEVLIHLEPEEERVLTRI
291aa_WRLP81    IDFHLVVSSSMTIESAHSLCDEIEAEIMNFYAKAEVLIHLEPEEERVLTRI
291aa_ScottA    IDFHLVVSSSMTIESAHSLCDEIEAEIMNFYAKAEVLIHLEPEEERVLTRI
                ***************************************************
```

Percent Identity Matrix

1: 291aa_WRLP46	100.00	100.00	100.00	100.00	99.66	99.66
2: 291aa_WRLP95	100.00	100.00	100.00	100.00	99.66	99.66
3: 291aa_WRLP85	100.00	100.00	100.00	100.00	99.66	99.66
4: 291aa_EGDe	100.00	100.00	100.00	100.00	99.66	99.66
5: 291aa_WRLP81	99.66	99.66	99.66	99.66	100.00	100.00
6: 291aa_ScottA	99.66	99.66	99.66	99.66	100.00	100.00

Reference Sequence: 626aa_WRLP46

```
626aa_WRLP46    MKDWMKQNWQFITTGISGILIVIGCLVGSDVGDFWTAIIFLSAFVIGGFEQAKEGIQATI
626aa_WRLP95    MKDWMKQNWQFITTGISGILIVIGCLVGSDVGDFWTAIIFLSAFVIGGFEQAKEGIQATI
626aa_EGDe      MKDWMKQNWQFITTGISGILIVIGCLVGSDVGDFWTAIIFLSAFVIGGFEQAKEGIQATI
626aa_WRLP85    MKDWMKQNWQFITTGISGILIVIGCLVGSDVGDFWTAVIFLSAFVIGGFEQAKEGIQATI
626aa_WRLP81    MKEWMKQNWQFITTGISGILIVIGCLVGSDVGDFWTAVIFLSAFVIGGFEQAKEGIQATI
626aa_Scott     MKEWMKQNWQFITTGISGILIVIGCLVGSDVGDFWTAVIFLSAFVIGGFEQAKEGIQATI
                **:***********************************:*********************

626aa_WRLP46    KTKKLNVELLMILAATGASIIGYWFEGAILIFIFSVSGALETYTTNKSKREITKLMAFQP
626aa_WRLP95    KTKKLNVELLMILAATGASIIGYWFEGAILIFIFSVSGALETYTTNKSKREITKLMAFQP
626aa_EGDe      KTKKLNVELLMILAATGASIIGYWFEGAILIFIFSVSGALETYTTNKSKREITKLMAFQP
626aa_WRLP85    KTKKLNVELLMILAATGASIIGYWFEGAILIFIFSVSGALETYTTNKSKREITKLMAFQP
626aa_WRLP81    KTKKLNVELLMILAATGASIIGYWFEGAILIFIFSVSGALETYTTNKSKREITKLMAFQP
626aa_Scott     KTKKLNVELLMILAATGASIIGYWFEGAILIFIFSVSGALETYTTNKSKREITKLMAFQP
                ************************************************************

626aa_WRLP46    ERAFRLLSNGDLEEVAAKELQLDDMVFVRPGESVPIDGVIVRGSTTLNEAAINGESVPAT
626aa_WRLP95    ERAFRLLSNGDLEEVAAKELQLDDMVFVRPGESVPIDGVIVRGSTTLNEAAINGESVPAT
626aa_EGDe      ERAFRLLSNGDLEEVAAKELQLDDMVFVRPGESVPIDGVIVRGSTTLNEAAINGESVPAT
626aa_WRLP85    ERAFRLLSNGDLEEVAAKELQLDDMVFVRPGESVPIDGVIVRGSTTLNEAAINGESVPAT
626aa_WRLP81    ERAFRLLPNGDLEEVAAKELQLDDMVFVRPGESVPIDGVIVRGSTTLNEAAINGESVPAT
626aa_Scott     ERAFRLLPNGDLEEVAAKELQLDDMVFVRPGESVPIDGVIVRGSTTLNEAAINGESVPAT
                *******.****************************************************

626aa_WRLP46    KTVGADVFGGTVNVSSAITVKVTQTFENTIFSKIIRLVETAQSEPSKTARFIERFEDVYV
626aa_WRLP95    KTVGADVFGGTVNVSSAITVKVTQTFENTIFSKIIRLVETAQSEPSKTARFIERFEDVYV
626aa_EGDe      KTVGADVFGGTVNVSSAITVKVTQTFENTIFSKIIRLVETAQSEPSKTARFIERFEDVYV
626aa_WRLP85    KTVGADVFGGTVNVSSAITVKVTQTFENTIFSKIIRLVETAQSEPSKTARFIERFEDVYV
626aa_WRLP81    KTVGADVFGGTVNVSSAITVKVTQTFDNTIFSKIIRLVESAQSEPSKTARFIERFEDAYV
626aa_Scott     KTVGADVFGGTVNVSSAITVKVTQTFDNTIFSKIIRLVESAQSEPSKTARFIERFEDAYV
                **************************:************:*****************.**

626aa_WRLP46    KAVLLFVLVMMFLPHFALGWSWNETFYRAMVLLTVASPCALVASVTPATLAAISNGARHG
626aa_WRLP95    KAVLLFVLVMMFLPHFALGWSWNETFYRAMVLLTVASPCALVASVTPATLAAISNGARHG
626aa_EGDe      KAVLLFVLVMMFLPHFALGWSWNETFYRAMVLLTVASPCALVASVTPATLAAISNGARHG
626aa_WRLP85    KAVLLFVLVMMFLPHFALGWSWNETFYRAMVLLTVASPCALVASVTPATLAAISNGARHG
626aa_WRLP81    KAVLLFVLVMMFLPHFALGWSWNETFYRAMVLLTVASPCALVASVTPATLAAISNGARHG
626aa_Scott     KAVLLFVLVMMFLPHFALGWSWNETFYRAMVLLTVASPCALVASVTPATLAAISNGARHG
                ************************************************************

626aa_WRLP46    ILFKGGVHLENLRGVKAIAFDKTGTLTNGTPALTDRLFAENVDKQLVINVVGAMERQSLH
626aa_WRLP95    ILFKGGVHLENLRGVKAIAFDKTGTLTNGTPALTDRLFAENVDKQLVINVVGAMERQSLH
626aa_EGDe      ILFKGGVHLENLRGVKAIAFDKTGTLTNGTPALTDRLFAENVDKQLVINVVGAMERQSLH
626aa_WRLP85    ILFKGGVHLENLRGVKAIAFDKTGTLTNGTPALTDRLFAENVDKQLVINVVGAMERQSLH
626aa_WRLP81    ILFKGGVHLENLRGVKAVAFDKTGTLTNGTPELTDRLFAENVDKQQVINVVGAMERQSLH
626aa_Scott     ILFKGGVHLENLRGVKAVAFDKTGTLTNGTPELTDRLFAENVDKQQVINVVGAMERQSLH
                *****************:************* ************* **************

626aa_WRLP46    PLAAAITQDLEPEITEKLTEIEVTDVPGWGVQAIYREGNWQVGKAGFVGKEAAAAFSNGA
626aa_WRLP95    PLAAAITQDLEPEITEKLTEIEVTDVPGWGVQAIYREGNWQVGKAGFVGKEAAAAFSNGA
626aa_EGDe      PLAAAITQDLEPEITEKLTEIEVTDVPGWGVQAIYREGNWQVGKAGFVGKEAAAAFSNGA
626aa_WRLP85    PLAAAITQDLEPEITEKLTEIEVTDVPGWGVQAIYREGNWQVGKAGFVGKEAAAAFSNGA
626aa_WRLP81    PLAAAITQDLEAEITEKLTEIEVTDVAGWGVQAIYQEGNWQVGKAGFVGKEAAAAFSNGA
626aa_Scott     PLAAAITQDLEAEITEKLTEIEVTDVAGWGVQAIYQEENWQVGKAGFVGEEAAAAFSNGA
                ***********.**************.********:* ***********:**********
```

```
626aa_WRLP46    FERLASEGKTIVYVAKDGVIQAMFALKDTCRPEAIRTIKALQAKGIKTIMVTGDNEQTGA
626aa_WRLP95    FERLASEGKTIVYVAKDGVIQAMFALKDTCRPEAIRTIKALQAKGIKTIMVTGDNEQTGA
626aa_EGDe      FERLASEGKTIVYVAKDGVIQAMFALKDTCRPEAIRTIKALQAKGIKTIMVTGDNEQTGA
626aa_WRLP85    FERLASEGKTIVYVAKDGVIQAMFALKDTCRPEAIRTIKALQAKGIKTIMVTGDNEQTGA
626aa_WRLP81    FERLAGEGKTIVYVAKDGVIQAMFALKDTCRPEAIRTIKALQAKGIKTIMVTGDNEQTGA
626aa_Scott     FERLAGEGKTIVYVAKDGVIQAMFALKDTCRPEAIRTIKALQAKGIKTIMVTGDNEQTGA
                *****.******************************************************

626aa_WRLP46    AIQAELGMDYVVSGCLPEKKVDVLRELSVTYGSVAMVGDGINDAPALAHAAVGIAMGEGT
626aa_WRLP95    AIQAELGMDYVVSGCLPEKKVDVLRELSVTYGSVAMVGDGINDAPALAHAAVGIAMGEGT
626aa_EGDe      AIQAELGMDYVVSGCLPEKKVDVLRELSVTYGSVAMVGDGINDAPALAHAAVGIAMGEGT
626aa_WRLP85    AIQAELGMDYVVSGCLPEKKVDVLRELSVTYGSVAMVGDGINDAPALAHAAVGIAMGEGT
626aa_WRLP81    AIQAELGMDYVVSGCLPEKKVDVLKELSVTYGSVAMVGDGINDAPALAHAAVGIAMGEGT
626aa_Scott     AIQAELGMDYVVSGCLPEKKVDVLKELSVTYGSVAMVGDGINDAPALAHAAVGIAMGEGT
                ************************:***********************************

626aa_WRLP46    DIAMETADVVLMKNDLEKIPYAYTLSERLHWITWQNICFAIAVILVLITANVFQLINLPF
626aa_WRLP95    DIAMETADVVLMKNDLEKIPYAYTLSERLHWITWQNICFAIAVILVLITANVFQLINLPF
626aa_EGDe      DIAMETADVVLMKNDLEKIPYAYTLSERLHWITWQNICFAIAVILVLITANVFQLINLPF
626aa_WRLP85    DIAMETADVVLMKNDLEKIPYAYTLSERLHWITWQNICFAIAVILVLITANVFQLINLPF
626aa_WRLP81    DIAMETADVVLMKNDLEKIPYAYNLSERLHWITWQNICFAIAVILVLITANVFQLINLPF
626aa_Scott     DIAMETADVVLMKNDLEKIPYAYNLSERLHWITWQNICFAIAVILALITANVFQLINLPF
                ***********************.*********************.**************

626aa_WRLP46    GVVGHEGSTILVILNGLRLLRSNRKK
626aa_WRLP95    GVVGHEGSTILVILNGLRLLRSNRKK
626aa_EGDe      GVVGHEGSTILVILNGLRLLRSNRKK
626aa_WRLP85    GVVGHEGSTILVILNGLRLLRSNRKK
626aa_WRLP81    GVVGHEGSTILVILNGLRLLRSNRKK
626aa_Scott     GVVGHEGSTILVILNGLRLLRSNRKK
                **************************
```

Percent Identity Matrix

1:	626aa_WRLP46	100.00	100.00	100.00	99.84	97.60	97.12
2:	626aa_WRLP95	100.00	100.00	100.00	99.84	97.60	97.12
3:	626aa_EGDe	100.00	100.00	100.00	99.84	97.60	97.12
4:	626aa_WRLP85	99.84	99.84	99.84	100.00	97.76	97.28
5:	626aa_WRLP81	97.60	97.60	97.60	97.76	100.00	99.52
6:	626aa_Scott	97.12	97.12	97.12	97.28	99.52	100.00

Reference Sequence: 627aa_WRLP46

```
627aa_WRLP46    MISYLIKSRQGQFLAIGILFAAAGFIFGTMNSEYSRWLFYAAIFFLGFYASKNAIVETVR
627aa_WRLP81    MISYLIKSRQGQFLAIGILFAAAGFIFGTMNSEYSRWLFYAAIFFLGFYASKNAIVETVR
627aa_WRLP95    MISYLIKSRQGQFLAIGILFAAAGFIFGTMNSEYSRWLFYAAIFFLGFYASKNAIVETVR
                ************************************************************

627aa_WRLP46    YKSPNVDLLMILAALGAVIFDFESEGAALLLIFAAAEVLEDYANNKSTSAISELMAQVPE
627aa_WRLP81    YKSPNVDLLMILAALGAVIFDFESEGAALLLIFAAAEVLEDYANNKSTSAISELMAQVPE
627aa_WRLP95    YKSPNVDLLMILAALGAVIFDFESEGAALLLIFAAAEVLEDYANNKSTSAISELMAQVPE
                ************************************************************

627aa_WRLP46    TAQVLKENGEVVTVPTEDLNVGERVVVSKGEQIPIDGIIDRKSIVNESALTGESVPVVKE
627aa_WRLP81    TAQVLKENGEVVTVPTEDLNVGERVVVSKGEQIPIDGIIDRKSIVNESALTGESVPVVKE
627aa_WRLP95    TAQVLKENGEVVTVPTEDLNVGERVVVSKGEQIPIDGIIDRKSIVNESALTGESVPVVKE
                ************************************************************

627aa_WRLP46    AEDEVFAGTINEGDVFYIDVTKSSDETVFSNIIRMVEEAQSRPSRISKFIDRIESKYVIS
627aa_WRLP81    AEDEVFAGTINEGDVFYIDVTKSSDETVFSNIIRMVEEAQSRPSRISKFIDRIESKYVIS
627aa_WRLP95    AEDEVFAGTINEGDVFYIDVTKSSDETVFSNIIRMVEEAQSRPSRISKFIDRIESKYVIS
                ************************************************************

627aa_WRLP46    VLVIVPIFIVVMYALMDLPFEEAFYRGMVFLTVASPCALVASATPATLSAISNGAKNGIL
627aa_WRLP81    VLVIVPIFIVVMYALMDLPFEEAFYRGMVFLTVASPCALVASATPATLSAISNGAKNGIL
627aa_WRLP95    VLVIVPIFIVVMYALMDLPFEEAFYRGMVFLTVASPCALVASATPATLSAISNGAKNGIL
                ************************************************************

627aa_WRLP46    FKGGAAMEALSTMDILYTDKTGTLTYGEFKVDEYSAPDDVLKEVIYMEQQSSHPIARAIV
627aa_WRLP81    FKGGAAMEALSTMDILYTDKTGTLTYGEFKVDEYSAPDDVLKEVIYMEQQSSHPIARAIV
627aa_WRLP95    FKGGAAMEALSTMDILYTDKTGTLTYGEFKVDEYSAPDDVLKEVIYMEQQSSHPIARAIV
                ************************************************************

627aa_WRLP46    TAFKETDLSSVDHNEPVSEIAGSGIKKGTVRVGKPSAFSTFKNYDRFKQYFQKGNTIILA
627aa_WRLP81    TAFKETDLSSVDHNEPVSEIAGSGIKKGTVRVGKPSAFSTFKNYDRFKQYFQKGNTIILA
627aa_WRLP95    TAFKETDLSSVDHNEPVSEIAGSGIKKGTVRVGKPSAFSTFKNYDRFKQYFQKGNTIILA
                ************************************************************

627aa_WRLP46    AKEEEVVGYFSLSDQIRRQSADAVANFQKEGIKVTLLTGDNEEVTETVAEVVGVDDYKAS
627aa_WRLP81    AKEEEVVGYFSLSDQIRRQSADAVANFQKEGIKVTLLTGDNEEVTETVAEVVGVDDYKAS
627aa_WRLP95    AKEEEVVGYFSLSDQIRRQSADAVANFQKEGIKVTLLTGDNEEVTETVAEVVGVDDYKAS
                ************************************************************

627aa_WRLP46    MLPEDKIAYVRESQDKEEVVGMIGDGINDAPALANADIGIAMGSGSSVAMESSDVVVVKN
627aa_WRLP81    MLPEDKIAYVRESQDKEEVVGMIGDGINDAPALANADIGIAMGSGSSVAMESSDVVVVKN
627aa_WRLP95    MLPEDKIAYVRESQDKEEVVGMIGDGINDAPALANADIGIAMGSGSSVAMESSDVVVVKN
                ************************************************************

627aa_WRLP46    DLSKLFYSYKLSKKLNKIILQNVIFSISVIVTLIVLNLFGVLGLPLAVLFHEGSTILVIL
627aa_WRLP81    DLSKLFYSYKLSKKLNKIILQNVIFSISVIVTLIVLNLFGVLGLPLAVLFHEGSTILVIL
627aa_WRLP95    DLSKLFYSYKLSKKLNKIILQNVIFSISVIVTLIVLNLFGVLGLPLAVLFHEGSTILVIL
                ************************************************************

627aa_WRLP46    NGLRLLGSKGPKQEERVSDPSLKSVKV
627aa_WRLP81    NGLRLLGSKGPKQEERVSDPSLKSVKV
627aa_WRLP95    NGLRLLGSKGPKQEERVSDPSLKSVKV
                ***************************
```

Percent Identity Matrix

```
     1: 627aa_WRLP46  100.00  100.00  100.00
     2: 627aa_WRLP81  100.00  100.00  100.00
     3: 627aa_WRLP95  100.00  100.00  100.00
```

Reference Sequence: 681aa_WRLP95

```
681aa_WRLP95     MSNNKKHQNHEHHSHQEHENHTSHGNHEHHHHGNFKSKFFISLIFAIPIIILSPMMGVKL
653aa_WRLP81     MS--------------------------------IKNRFIIGVIGSVPLLI-----NMFM
                 **                                :*.:*:*.:* ::*::*     .: :

681aa_WRLP95     PFQISFTGSD----WIVLILATILFFYGGKPFLSGAKDEISTKKPGMMTLVALGISVAYI
653aa_WRLP81     SFGGSMLGGDKYGVWILFAFGSLVYWFSGLPFLRTAVASFKNHHANMDTLVGLGTTIAYV
                 .*  *: *.*    **:: :.:::::.* ***  *  .:..::..* ***.** ::**:

681aa_WRLP95     YSLYAFYMNNFSGSSTHTMDFFWELATLILIMLLGHWIEMNAVGNAGNALKKMAELLPNT
653aa_WRLP81     YSLYAMF-------ARPNETYFEAVAVVITLILLGSYFEERMKASASSAVDKLMGLQAKD
                 *****::       :  .  :*  :*.:* ::***  ::* .   ..*..*:.*:  * .:

681aa_WRLP95     AVKLIDNNQREEVKISDIHIDDIVEVRAGESIPTDGIIVQGETSIDESLVTGESKKVHKT
653aa_WRLP81     A-EVLRDGEFIKLPIEEIIVGDLIRVKPGEKVAVDGQIVEGTSTLDESMVTGESMPVEKG
                 * ::: :.:  :: *.:* :.*::.*:.**.:..** **:* :::***:*****  *.*

681aa_WRLP95     HNDDVIGGSINGSGTVQVKVTATGENGYLSQVMGLVNQAQNDKSKAELLSDKVAGYLFYF
653aa_WRLP81     PGDNVIGATLNNTGSFTFEVTKVGADTMLSNIAEMVRHAQNSRAPIQKTVDRISNIFVPI
                  .*:***.::*.:*:. .:** .* :  **::  :*.:***.::  :   *:::. :. :

681aa_WRLP95     AVSIGLISFIVW-MLIQNNVDFALERLVTVLVIACPHALGLAIPLVTARSTSIGAHNGLI
653aa_WRLP81     VLMISILTFIVWYVFLGSTLVTAMIFSVSVMIIACPCALGIATPTALMVGTGRSAKLGIL
                 .: *.::**** ::: ..:  *:   *:*::**** ***:* * .   .*. .*: *::

681aa_WRLP95     IKNRESVEIAQHIDYIMMDKTGTLTEGNFSVNHYESFTDELNNEEILSLFASLESNSNHP
653aa_WRLP81     IKNAEVLEATHDIKTVVMDKTGTITVGKPQVTDIISI-GRISENEILRIAAGLEDSSEHP
                 *** * :* ::.*. ::******:* *: .*..  *: ..:.::*** : *.**..*:**

681aa_WRLP95     LATGIVDFAKGKNISYATPQEVNNIPGVGLEGTVDNKKLKIVNVSYLDKSNFDYN-----
653aa_WRLP81     LALAVINEAKDKKITPAVAKNFTAISGKGVQALIDGKQAFIGN----DRLSDDFNMTDDL
                 ** .::: **.*:*: *..::.. *.* *::. :*.*:  * *    *: . *:*

681aa_WRLP95     KEQFTNLAQQGNSISYLIHDRQVIGIIAQGDKIKESSKQMVSDLLSRNITPVMLTGDNKE
653aa_WRLP81     KVKMTSLQAQAKTVVLVGYDGQIIALIGIQDAPKSSSKAAIRAMQKSGFHTVMLTGDNRL
                 * ::*.*  *.:::  : :* *:*.:*.  *  *.***  :  : . .: .*******:

681aa_WRLP95     VAQTVAEELGISDVHAQLMPEDKESIIQDYQSNGSKIMMVGDGINDAPSLIRADIGMAIG
653aa_WRLP81     VAQAIADDIGIDEVIADVMPGDKAQHIRKLQEKGA-VAFVGDGINDAPALSTATVGIAMG
                 ***::*:::**.:* *::** ** . *:. *.:*: : :*********:*  * :*:*:*

681aa_WRLP95     AGTDVAIESGDVILVKSNPSDIINFLSLSKNTMKKMVQNLWWGAGYNVIAVPLAAGILAS
653aa_WRLP81     SGSDIAIESGGIVLVKNDLMDVVTSLVLARKTYSRILINLFWAFIYNVIGIPVAAGIFSA
                 :*:*:*****.::***.:  *::. * *:::* .::: **:*.  ****.:*:****:::

681aa_WRLP95     IGLILSPAVGAILMSLSTIIVAINAFTLKL-------------K
653aa_WRLP81     LGFTLSPELAGLAMALSSITVVLSSLLLNYVRLPKSSETLIGNS
                 :*: *** :..: *:**:* *.:.:: *:              .
```

Percent Identity Matrix

```
     1: 681aa_WRLP95  100.00   36.19
     2: 653aa_WRLP81   36.19  100.00
```

Reference Sequence: 737aa_WRLP64

```
737aa_WRLP46    MSDKYVRQDLNVFGMTCAACSTRIEKSLNKADGVEKANVNLVTENAAVYYDPEVTSTEDL
737aa_WRLP95    MSDKYVRQDLNVFGMTCAACSTRIEKSLNKADGVEKANVNLVTENAAVYYDPEVTSTEDL
737aa_EGDe      MSDKYVRQDLNVFGMTCAACSTRIEKSLNKADGVEKANVNLVTENAAVYYDPEVTSTEDL
737aa_WRLP85    MSDKYVRQDLNVFGMTCAACSTRIEKSLNKADGVEKANVNLVTENAAVYYDPEVTSTEDL
737aa_WRLP81    MSDKYVRQDLNIFGMTCAACSTRIEKSLNKADGVEKANVNLVTENAAVYYDPEVTSTEDL
737aa_ScottA    MSDKYVRQDLNIFGMTCAACSTRIEKSLNKADGVEKANVNLVTENAAVYYDPEVTSTEDL
                ***********:************************************************

737aa_WRLP46    IKVVKHAGYDAAEKMSKEEKDAVLEKNFKKEVRRFILSALLSLPLLLTMVTHIPYIHEMA
737aa_WRLP95    IKVVKHAGYDAAEKMSKEEKDAVLEKNFKKEVRRFILSALLSLPLLLTMVTHIPYIHEMA
737aa_EGDe      IKVVKHAGYDAAEKMSKEEKDAVLEKNFKKEVRRFILSALLSLPLLLTMVTHIPYIHEMA
737aa_WRLP85    IKVVKHAGYDAAEKMSKEEKDAVLEKNFKKEVRRFILSAVLSLPLLLTMVTHIPYIHEMA
737aa_WRLP81    IKVVKHAGYDAAEKMSKEEKDAVLEKNFKKEVRRFILSAVLSLPLLLTMVTHIPYIHEMV
737aa_ScottA    IKVVKHAGYDAAEKMSKEEKDAVLEKNFKKEVRRFILSAVLSLPLLLTMVTHIPYIHEMV
                ***************************************:*******************.

737aa_WRLP46    FAETIGNWINPTIQLVLATIVQFYIGWRFYDGAYKALRGKSANMDVLVALGTSAAYFYSV
737aa_WRLP95    FAETIGNWINPTIQLVLATIVQFYIGWRFYDGAYKALRGKSANMDVLVALGTSAAYFYSV
737aa_EGDe      FAETIGNWINPTIQLVLATIVQFYIGWRFYDGAYKALRGKSANMDVLVALGTSAAYFYSV
737aa_WRLP85    FAETIGNWINPTIQLVLATLVQFYIGWRFYDGAYKALRGKSANMDVLVALGTSAAYFYSV
737aa_WRLP81    FAETIGNWINPTIQLVLATIVQFYIGWRFYDGAYKALRGKSANMDVLVALGTSAAYFYSV
737aa_ScottA    FAETIGNWINPTIQLVLATIVQFYIGWRFYDGAYKALRGKSANMDVLVALGTSAAYFYSV
                *******************:****************************************

737aa_WRLP46    VEYVRHIIDPSVMPHYYFETSAVLITLILLGKLLESYATSRTTESIAGLLELQAKEATVI
737aa_WRLP95    VEYVRHIIDPSVMPHYYFETSAVLITLILLGKLLESYATSRTTESIAGLLELQAKEATVI
737aa_EGDe      VEYVRHIIDPSVMPHYYFETSAVLITLILLGKLLESYATSRTTESIAGLLELQAKEATVI
737aa_WRLP85    VEYVRHIIDPSVMPHYYFETSAVLITLILLGKLLESYATSRTTESIAGLLELQAKEATVI
737aa_WRLP81    VEYIRHMIDPSVMPHYYFETSAVLITLILLGKLLESYATSRTTESIAGLLELQAKEATVI
737aa_ScottA    VEYIRHMIDPSVMPHYYFETSAVLITLILLGKLLESYATSRTTESIAGLLELQAKEATVI
                ***:**:*****************************************************

737aa_WRLP46    REGKEWLVPVDSLKIGDVILVRPGEKVPMDAEIISGETSIDEAMITGEPVPVEKKPGDSV
737aa_WRLP95    REGKEWLVPVDSLKIGDVILVRPGEKVPMDAEIISGETSIDEAMITGEPVPVEKKPGDSV
737aa_EGDe      REGKEWLVPVDSLKIGDVILVRPGEKVPMDAEIISGETSIDEAMITGEPVPVEKKPGDSV
737aa_WRLP85    REGKEWLVPVDSLKIGDVILVRPGEKVPMDAEIISGETSIDEAMITGEPVPVEKKPGDSV
737aa_WRLP81    REGKEWLVPVDSLKIGDIILVRPGEKVPMDAEIISGETSIDEAMITGEPVPVEKKPGDSV
737aa_ScottA    REGKEWLVPVDSLKIGDIILVRPGEKVPMDAEIISGETSIDEAMITGEPVPVEKKPGDSV
                *****************:******************************************

737aa_WRLP46    IGATINFDGAFQAKITKRMEETVLESIIRLVEEAQGIKAPIQRLADRISGIFVPIVLGIA
737aa_WRLP95    IGATINFDGAFQAKITKRMEETVLESIIRLVEEAQGIKAPIQRLADRISGIFVPIVLGIA
737aa_EGDe      IGATINFDGAFQAKITKRMEETVLESIIRLVEEAQGIKAPIQRLADRISGIFVPIVLGIA
737aa_WRLP85    IGATINFDGAFQAKITKRMEETVLESIIRLVEEAQGIKAPIQRLADRISGIFVPIVLGIA
737aa_WRLP81    IGATINFDGAFQAKITKRMEETVLESIIRLVEEAQGIKAPIQRLADRISGIFVPIVLGIA
737aa_ScottA    IGATINFDGAFQAKITKRMEETVLESIIRLVEEAQGIKAPIQRLADRISGIFVPIVLGIA
                ************************************************************

737aa_WRLP46    AVTFIIWYLVTGTVDGSLEAAIAVLVIACPCALGLATPTAIMAGTGKGAESGILFKGGEH
737aa_WRLP95    AVTFIIWYLVTGTVDGSLEAAIAVLVIACPCALGLATPTAIMAGTGKGAESGILFKGGEH
737aa_EGDe      AVTFIIWYLVTGTVDGSLEAAIAVLVIACPCALGLATPTAIMAGTGKGAESGILFKGGEH
737aa_WRLP85    AVTFIIWYLVTGTVDGSLEAAIAVLVIACPCALGLATPTAIMAGTGKGAESGILFKGGEH
737aa_WRLP81    AVTFIIWYLVTGTVDGSLEAAIAVLVIACPCALGLATPTAIMAGTGKGAESGILFKGGEH
737aa_ScottA    AVTFIIWYLVTGTVDGSLEAAIAVLVIACPCALGLATPTAIMAGTGKGAESGILFKGGEH
                ************************************************************
```

```
737aa_WRLP46    LERTSKVDTIVFDKTGTLTEGKLEVSDKKAANDQFFPYLFLMEQQSEHPIAKAIIKMLEP
737aa_WRLP95    LERTSKVDTIVFDKTGTLTEGKLEVSDKKAANDQFFPYLFLMEQQSEHPIAKAIIKMLEP
737aa_EGDe      LERTSKVDTIVFDKTGTLTEGKLEVSDKKAANDQFFPYLFLMEQQSEHPIAKAIIKMLEP
737aa_WRLP85    LERTSKVDTIVFDKTGTLTEGKLEVSDKKAANDHFFPYLFLMEQQSEHPIAKAIIKMLEP
737aa_WRLP81    LERTSKVDTIVFDKTGTLTEGKLEVSDKKAANDHFFPYLFLMEQQSEHPIAKAIIKMLEP
737aa_ScottA    LERTSKVDTIVFDKTGTLTEGKLEVSDKKAANDHFFPYLFLMEQQSEHPIAKAIIKMLEP
                *********************************:**************************

737aa_WRLP46    ENMDVSAIKQGKIRAKAGHGMTGNLDDSKVELGAYRYVSSLTTIPKEDDELIESWMHAGK
737aa_WRLP95    ENMDVSAIKQGKIRAKAGHGMTGNLDDSKVELGAYRYVSSLTTIPKEDDELIESWMHAGK
737aa_EGDe      ENMDVSAIKQGKIRAKAGHGMTGNLDDSKVELGAYRYVSSLTTIPKEDDELIESWMHAGK
737aa_WRLP85    ENMDVSAIKQGKIRAKAGHGMTGNLDDSKVELGAYRYVSSLTTIPKEDDELIESWMHAGK
737aa_WRLP81    ENIDASAVKQGKIRAKAGHGMTGNLDDSKVELGAYRYVSSLTTIPKEDDELIESWMHAGK
737aa_ScottA    ENIDASAVKQGKIRAKAGHGMTGNLDDSKVELGAYRYVSSLTTIPKEDDELIESWMHAGK
                **:*.**:****************************************************

737aa_WRLP46    TVVAMAIDGVYAGALALSDTPRPEAKEAIQKLKAQGIKTAICSGDQSVVVENMAKDLGID
737aa_WRLP95    TVVAMAIDGVYAGALALSDTPRPEAKEAIQKLKAQGIKTAICSGDQSVVVENMAKDLGID
737aa_EGDe      TVVAMAIDGVYAGALALSDTPRPEAKEAIQKLKAQGIKTAICSGDQSVVVENMAKDLGID
737aa_WRLP85    TVVAMAIDGVYAGALALSDTPRPEAKEAIQKLKAQGIKTAICSGDQSVVVENMAKDLGID
737aa_WRLP81    TVVAMAIDGVYAGALALSDTPRPEAKEAIQKLKAQGIKTAICSGDQSVVVENMAKDLGID
737aa_ScottA    TVVAMAIDGVYAGALALSDTPRPEAKEAIQKLKAQGIKTAICSGDQSVVVENMAKDLGID
                ************************************************************

737aa_WRLP46    MFFAEQLPNDKSALVEKLQQDGHIVAFVGDGINDAPALAASDIGISIGTGTDIAIETGDV
737aa_WRLP95    MFFAEQLPNDKSALVEKLQQDGHIVAFVGDGINDAPALAASDIGISIGTGTDIAIETGDV
737aa_EGDe      MFFAEQLPNDKSALVEKLQQDGHIVAFVGDGINDAPALAASDIGISIGTGTDIAIETGDV
737aa_WRLP85    MFFAEQLPNDKSALVEKLQQDGHIVAFVGDGINDAPALAASDIGISIGTGTDIAIETGDV
737aa_WRLP81    MFFAEQLPNDKSALVEKLQQDGHIVAFVGDGINDAPALAASDIGISIGTGTDIAIETGDV
737aa_ScottA    MFFAEQLPNDKSALVEKLQQDGHIVAFVGDGINDAPALAASDIGISIGTGTDIAIETGDV
                ************************************************************

737aa_WRLP46    TLVSHRLTLIPETIELSKATMRNIRQNFFWALAYNCAGIPIAALGLLAPWVAGLAMAFSS
737aa_WRLP95    TLVSHRLTLIPETIELSKATMRNIRQNFFWALAYNCAGIPIAALGLLAPWVAGLAMAFSS
737aa_EGDe      TLVSHRLTLIPETIELSKATMRNIRQNFFWALAYNCAGIPIAALGLLAPWVAGLAMAFSS
737aa_WRLP85    TLVSHRLTLIPETIELSKATMRNIRQNFFWALAYNCAGIPIAALGLLAPWVAGLAMAFSS
737aa_WRLP81    TLVSHRLTLIPETIELSKATMRNIRQNFFWALAYNCAGIPIAALGLLAPWVAGLAMAFSS
737aa_ScottA    TLVSHRLTLIPETIELSKATMRNIRQNFFWALAYNCAGIPIAALGLLAPWVAGLAMAFSS
                ************************************************************

737aa_WRLP46    VSVVTNALRLKRYKFKS
737aa_WRLP95    VSVVTNALRLKRYKFKS
737aa_EGDe      VSVVTNALRLKRYKFKS
737aa_WRLP85    VSVVTNALRLKRYKFKS
737aa_WRLP81    VSVVTNALRLKRYKFKS
737aa_ScottA    VSVVTNALRLKRYKFKS
                *****************
```

Percent Identity Matrix

```
     1: 737aa_WRLP46   100.00  100.00  100.00   99.59   98.64   98.64
     2: 737aa_WRLP95   100.00  100.00  100.00   99.59   98.64   98.64
     3: 737aa_EGDe     100.00  100.00  100.00   99.59   98.64   98.64
     4: 737aa_WRLP85    99.59   99.59   99.59  100.00   98.78   98.78
     5: 737aa_WRLP81    98.64   98.64   98.64   98.78  100.00  100.00
     6: 737aa_ScottA    98.64   98.64   98.64   98.78  100.00  100.00
```

Reference Sequence: CadA1_WRLP46

```
CadA1_WRLP46    MAEK---TVYRVDGLSCTNCAAKFERNVKEIEGVTEAIVNFGASKITVTGEAS---IQQV
CadA1_WRLP76    MAEK---TVYRVDGLSCTNCAAKFERNVKEIEGVTEAIVNFGASKITVTGEAS---IQQV
CadA1_WRLP22    MAEK---TVYRVDGLSCTNCAAKFERNVKEIEGVTEAIVNFGASKITVTGEAS---IQQV
CadA1_WRLP13    MAEK---TVYRVDGLSCTNCAAKFERNVKEIEGVTEAIVNFGASKITVTGEAS---IQQV
CadA1_WRLP14    MAEK---TVYRVDGLSCTNCAAKFERNVKEIEGVTEAIVNFGASKITVTGEAS---IQQV
CadA1_WRLP51    MAEK---TVYRVDGLSCTNCAAKFERNVKEIEGVTEAIVNFGASKITVTGEAS---IQQV
CadA1_WRLP95    MAEK---TVYRVDGLSCTNCAAKFERNVKEIEGVTEAIVNFGASKITVTGEAS---IQQV
CadA1_WRLP79    MAEK---TVYRVDGLSCTNCAAKFERNVKEIEGVTEAIVNFGASKITVTGEAS---IQQV
CadA2_WRLP81    MSEKMTEKTYRIEGLSCTNCAGKFEKNVKQLPGVTSATVNFGASRISVEGQTT---IEEL
CadA3_EGDe      MSKASKQTTYRVDGMSCTNCAGKFEKNVKNLEGVTDAKVNFGAGKISVYGETS---ISQI
CadA4_ScottA    MKKE-----YILEGLTCANCAGKIENDVKKISGIENVTLNLMNTTLAFDKKDDNGLDEEI
                * :      * ::*::*:***.*:*.:**:: *: .. :*:    ::.  :      .::

CadA1_WRLP46    EQ-AGAFE-HLKIIPEKESFTDPEH---FTDHQSFIRKNWRLLLSGLFIAVGYASQIMNG
CadA1_WRLP76    EQ-AGAFE-HLKIIPEKESFTDPEH---FTDHQSFIRKNWRLLLSGLFIAVGYASQIMNG
CadA1_WRLP22    EQ-AGAFE-HLKIIPEKESFTDPEH---FTDHQSFIRKNWRLLLSGLFIAVGYASQIMNG
CadA1_WRLP13    EQ-AGAFE-HLKIIPEKESFTDPEH---FTDHQSFIRKNWRLLLSGLFIAVGYASQIMNG
CadA1_WRLP14    EQ-AGAFE-HLKIIPEKESFTDPEH---FTDHQSFIRKNWRLLLSGLFIAVGYASQIMNG
CadA1_WRLP51    EQ-AGAFE-HLKIIPEKESFTDPEH---FTDHQSFIRKNWRLLLSGLFIAVGYASQIMNG
CadA1_WRLP95    EQ-AGAFE-HLKIIPEKESFTDPEH---FTDHQSFIRKNWRLLLSGLFIAVGYASQIMNG
CadA1_WRLP79    EQ-AGAFE-HLKIIPEKESFTDPEH---FTDHQSFIRKNWRLLLSGLFIAVGYASQIMNG
CadA2_WRLP81    EE-AGAFE-NLIIRDDQENDEQVRS------KESFIKRNIALIISLSFILVAVISQLSLG
CadA3_EGDe      EK-AGAFE-NLRVTDEKDYSSKPAK------KESFLKKNWHLVVSIIFIILAFISQNISG
CadA4_ScottA    EKIVHTYEPEVGVFPKQEYKKEPTKNLSLKDNKIFLR----LVVGAIILIFAVIANMQDG
                *: . ::* .: :  .::   .         :: *::    *::.  ::  ..  ::   *

CadA1_WRLP46    EDFYLTNALFIFAIFIGGYSLFKEGFKNLLKFE-FTMETLMTIAIIGAAFIGEWAEGSIV
CadA1_WRLP76    EDFYLTNALFIFAIFIGGYSLFKEGFKNLLKFE-FTMETLMTIAIIGAAFIGEWAEGSIV
CadA1_WRLP22    EDFYLTNALFIFAIFIGGYSLFKEGFKNLLKFE-FTMETLMTIAIIGAAFIGEWAEGSIV
CadA1_WRLP13    EDFYLTNALFIFAIFIGGYSLFKEGFKNLLKFE-FTMETLMTIAIIGAAFIGEWAEGSIV
CadA1_WRLP14    EDFYLTNALFIFAIFIGGYSLFKEGFKNLLKFE-FTMETLMTIAIIGAAFIGEWAEGSIV
CadA1_WRLP51    EDFYLTNALFIFAIFIGGYSLFKEGFKNLLKFE-FTMETLMTIAIIGAAFIGEWAEGSIV
CadA1_WRLP95    EDFYLTNALFIFAIFIGGYSLFKEGFKNLLKFE-FTMETLMTIAIIGAAFIGEWAEGSIV
CadA1_WRLP79    EDFYLTNALFIFAIFIGGYSLFKEGFKNLLKFE-FTMETLMTIAIIGAAFIGEWAEGSIV
CadA2_WRLP81    EDHLLTKALYILAIIIGGFDLFKEGFSDLIKLD-FSMESLMTIAIIGAAFIGEWAEGSIV
CadA3_EGDe      EDSTTTIILYVIAIVVGGFNLFKEGFANLIKLD-FTMESLMTIAIIGASIIGEWAEGSIV
CadA4_ScottA    INDWLKLALFLISYAILGGDVLLKAIRNIFKGQVFDENFLMSIATIGAFIIGETAEAVAV
                 :   .  *::::  : * .:: :.: :::* : *  : **:** *** :*** **.  *

CadA1_WRLP46    VILFAVSEALERYSMDKARQSIRSLMDIAPKEALVRRSGTDRMVHVDDIQIGDIMIIKPG
CadA1_WRLP76    VILFAVSEALERYSMDKARQSIRSLMDIAPKEALVRRSGTDRMVHVDDIQIGDIMIIKPG
CadA1_WRLP22    VILFAVSEALERYSMDKARQSIRSLMDIAPKEALVRRSGTDRMVHVDDIQIGDIMIIKPG
CadA1_WRLP13    VILFAVSEALERYSMDKARQSIRSLMDIAPKEALVRRSGTDRMVHVDDIQIGDIMIIKPG
CadA1_WRLP14    VILFAVSEALERYSMDKARQSIRSLMDIAPKEALVRRSGTDRMVHVDDIQIGDIMIIKPG
CadA1_WRLP51    VILFAVSEALERYSMDKARQSIRSLMDIAPKEALVRRSGTDRMVHVDDIQIGDIMIIKPG
CadA1_WRLP95    VILFAVSEALERYSMDKARQSIRSLMDIAPKEALVRRSGTDRMVHVDDIQIGDIMIIKPG
CadA1_WRLP79    VILFAVSEALERYSMDKARQSIRSLMDIAPKEALVRRSGTDRMVHVDDIQIGDIMIIKPG
CadA2_WRLP81    VILFAISEALERFSMDKARQSIRSLMDIAPKEALIRRNNVEQLVSVDKIDIDDIMIIKPG
CadA3_EGDe      VILFAFSEVLERYSMDKARQSIRSLMDIAPKEALIRRDDVEQMIAVSDIQIGDIMIIKPG
CadA4_ScottA    MLFYQIGELFQDIAVKRSKKSITDLMDIRPDYANLKVGNDIKKVKPETIKIGDIIIVKAG
                :::: ..* ::  ::.::::** .**** *. * :: ..  : :  . *.*.**:*:*.*
```

```
CadA1_WRLP46    QKIAMDGHVVKGYSAVNQAAITGESIPVEKNIDDSVFAGTLNEEGLLEVAVTKRVEDTTI
CadA1_WRLP76    QKIAMDGHVVKGYSAVNQAAITGESIPVEKNIDDSVFAGTLNEEGLLEVAVTKRVEDTTI
CadA1_WRLP22    QKIAMDGHVVKGYSAVNQAAITGESIPVEKNIDDSVFAGTLNEEGLLEVAVTKRVEDTTI
CadA1_WRLP13    QKIAMDGHVVKGYSAVNQAAITGESIPVEKNIDDSVFAGTLNEEGLLEVAVTKRVEDTTI
CadA1_WRLP14    QKIAMDGHVVKGYSAVNQAAITGESIPVEKNIDDSVFAGTLNEEGLLEVAVTKRVEDTTI
CadA1_WRLP51    QKIAMDGHVVKGYSAVNQAAITGESIPVEKNIDDSVFAGTLNEEGLLEVAVTKRVEDTTI
CadA1_WRLP95    QKIAMDGHVVKGYSAVNQAAITGESIPVEKNIDDSVFAGTLNEEGLLEVAVTKRVEDTTI
CadA1_WRLP79    QKIAMDGHVVKGYSAVNQAAITGESIPVEKNIDDSVFAGTLNEEGLLEVAVTKRVEDTTI
CadA2_WRLP81    QKIAMDGLVINGHSSVNQAAITGESVPVEKQLDDEVFAGTLNEEGVLEVKVTKKVTDTTI
CadA3_EGDe      QKIAMDGVVIKGYSAINQSAITGESIPVEKKVDDEVFAGTLNEEGLLEVKVTKHVEDTTI
CadA4_ScottA    EKVSLDGIVVAGESLLDTKALTGESVPRKTKTGDNVLSGCINQSGVLTIEVTKTFGESTV
                :*:::** *: * * ::  *:****:* :.: .*.*::* :*:.*:* : *** . ::*:

CadA1_WRLP46    SKIIHLVEEAQGERAPAQAFVDTFAKYYTPAIIVIAALIATVPPLLFGGNWETWVYQGLS
CadA1_WRLP76    SKIIHLVEEAQGERAPAQAFVDTFAKYYTPAIIVIAALIATVPPLLFGGNWETWVYQGLS
CadA1_WRLP22    SKIIHLVEEAQGERAPAQAFVDTFAKYYTPAIIVIAALIATVPPLLFGGNWETWVYQGLS
CadA1_WRLP13    SKIIHLVEEAQGERAPAQAFVDTFAKYYTPAIIVIAALIATVPPLLFGGNWETWVYQGLS
CadA1_WRLP14    SKIIHLVEEAQGERAPAQAFVDTFAKYYTPAIIVIAALIATVPPLLFGGNWETWVYQGLS
CadA1_WRLP51    SKIIHLVEEAQGERAPAQAFVDTFAKYYTPAIIVIAALIATVPPLLFGGNWETWVYQGLS
CadA1_WRLP95    SKIIHLVEEAQGERAPAQAFVDTFAKYYTPAIIVIAALIATVPPLLFGGNWETWVYQGLS
CadA1_WRLP79    SKIIHLVEEAQGERAPAQAFVDTFAKYYTPAIIVIAALIATVPPLLFGGNWETWVYQGLS
CadA2_WRLP81    AKIIHLVEEAQGERAPAQAFVDKFAKYYTPFIIIMALLIVVVPPLFFGGDWNKWLYQGLS
CadA3_EGDe      SKIIHLVEEAQGERAPAQAFVDKFAKYYTPTIMLIALLVVVVPPLFFGGDWDTWVYQGLS
CadA4_ScottA    AKIIDLVENASSKKAPTENFITKFSRYYTPVVVIVATLLAVIPPLFFGGEWSDWVNRGLI
                :***.***:*..::**:: *: .*::**** ::::* *:..:***:***:*. *: :** 

CadA1_WRLP46    VLVVGCPCALVVSTPVAIVTAIGNAAKNGVLVKGGVYLEEIGGLKAIAFDKTGTLTKGVP
CadA1_WRLP76    VLVVGCPCALVVSTPVAIVTAIGNAAKNGVLVKGGVYLEEIGGLKAIAFDKTGTLTKGVP
CadA1_WRLP22    VLVVGCPCALVVSTPVAIVTAIGNAAKNGVLVKGGVYLEEIGGLKAIAFDKTGTLTKGVP
CadA1_WRLP13    VLVVGCPCALVVSTPVAIVTAIGNAAKNGVLVKGGVYLEEIGGLKAIAFDKTGTLTKGVP
CadA1_WRLP14    VLVVGCPCALVVSTPVAIVTAIGNAAKNGVLVKGGVYLEEIGGLKAIAFDKTGTLTKGVP
CadA1_WRLP51    VLVVGCPCALVVSTPVAIVTAIGNAAKNGVLVKGGVYLEEIGGLKAIAFDKTGTLTKGVP
CadA1_WRLP95    VLVVGCPCALVVSTPVAIVTAIGNAAKNGVLVKGGVYLEEIGGLKAIAFDKTGTLTKGVP
CadA1_WRLP79    VLVVGCPCALVVSTPVAIVTAIGNAAKNGVLVKGGVYLEEIGGLKAIAFDKTGTLTKGVP
CadA2_WRLP81    ILVVGCPCSLVISTPVSIVSAIGNAAKNGVLVKGGVYLEEIGHLRAIAFDKTGTLTKGKP
CadA3_EGDe      LLVVGCPCSLVISTPVSIVSAIGNSAKNGVLVKGGIYLEEIGGLQAIAFDKTGTLTKGKP
CadA4_ScottA    FLVISCPCALVVSIPLGFFGGIGGASKHGILVKGSNFLEALNNVDTIVFDKTGTLTEGVF
                .**:.***:**:* *:.:. .**.::*:*:****. :** :. : :*.********:*

CadA1_WRLP46    VVTDYIELTEATNIQHNKNYI-IMAALEQLSQHPLASAIIKYGETREMDLTSINVNDFTS
CadA1_WRLP76    VVTDYIELTEATNIQHNKNYI-IMAALEQLSQHPLASAIIKYGETREMDLTSINVNDFTS
CadA1_WRLP22    VVTDYIELTEATNIQHNKNYI-IMAALEQLSQHPLASAIIKYGETREMDLTSINVNDFTS
CadA1_WRLP13    VVTDYIELTEATNIQHNKNYI-IMAALEQLSQHPLASAIIKYGETREMDLTSINVNDFTS
CadA1_WRLP14    VVTDYIELTEATNIQHNKNYI-IMAALEQLSQHPLASAIIKYGETREMDLTSINVNDFTS
CadA1_WRLP51    VVTDYIELTEATNIQHNKNYI-IMAALEQLSQHPLASAIIKYGETREMDLTSINVNDFTS
CadA1_WRLP95    VVTDYIELTEATNIQHNKNYI-IMAALEQLSQHPLASAIIKYGETREMDLTSINVNDFTS
CadA1_WRLP79    VVTDYIELTEATNIQHNKNYI-IMAALEQLSQHPLASAIIKYGETREMDLTSINVNDFTS
CadA2_WRLP81    VVTDFIATSSETD----INYLSIISSLESLSQHPLASAILNEADKTNVDYKSIQIEDFQS
CadA3_EGDe      VVTDFIPYSEHMD---EQNSLSIITALETMSQHPLASAIISKAMIDNVDYKSIEIDNFSS
CadA4_ScottA    EVTS-INTSNGFT---EEQLIEYGAKAETLSNHPIAMSI-KKAYGKEIEHS--ELDSYQE
                 **. *  :.        : :   :  * :*:**:* :* . .   ::: .  :::.: .
```

```
CadA1_WRLP46    ITGKGIRGTVDGNTYYVGSPVLFKELLASQFTDSIHRQVSDLQLKGKTAMLFGTNQKLIS
CadA1_WRLP76    ITGKGIRGTVDGNTYYVGSPVLFKELLASQFTDSIHRQVSDLQLKGKTAMLFGTNQKLIS
CadA1_WRLP22    ITGKGIRGTVDGNTYYVGSPVLFKELLASQFTDSIHRQVSDLQLKGKTAMLFGTNQKLIS
CadA1_WRLP13    ITGKGIRGTVDGNTYYVGSPVLFKELLASQFTDSIHRQVSDLQLKGKTAMLFGTNQKLIS
CadA1_WRLP14    ITGKGIRGTVDGNTYYVGSPVLFKELLASQFTDSIHRQVSDLQLKGKTAMLFGTNQKLIS
CadA1_WRLP51    ITGKGIRGTVDGNTYYVGSPVLFKELLASQFTDSIHRQVSDLQLKGKTAMLFGTNQKLIS
CadA1_WRLP95    ITGKGIRGTVDGNTYYVGSPVLFKELLASQFTDSIHRQVSDLQLKGKTAMLFGTNQKLIS
CadA1_WRLP79    ITGKGIRGTVDGNTYYVGSPVLFKELLASQFTDSIHRQVSDLQLKGKTAMLFGTNQKLIS
CadA2_WRLP81    ITGKGLTGIHQNIRYYIGSPKLFSASVIE--ETAVKVQYRQFQEQGKTAMYFGTDEQILG
CadA3_EGDe      ITGKGVKGEVNGITYYIGSSKLFESSLEK--SQSISQTYQSLQKQGKTAMLFGTESNILA
CadA4_ScottA    IAGHGISVLIKEKPVLAGNEKLMKQHNIEYMSNS----------ETGTVVYIAIDNVFAG
                *:*:*:    .      *.  *:.    .    :          :  *.: :. :. : .

CadA1_WRLP46    IVAVADEVRSSSQHVIKRLHELGIEKTIMLTGDNQATAQAIGQQVGVSEIEGELMPQDKL
CadA1_WRLP76    IVAVADEVRSSSQHVIKRLHELGIEKTIMLTGDNQATAQAIGQQVGVSEIEGELMPQDKL
CadA1_WRLP22    IVAVADEVRSSSQHVIKRLHELGIEKTIMLTGDNQATAQAIGQQVGVSEIEGELMPQDKL
CadA1_WRLP13    IVAVADEVRSSSQHVIKRLHELGIEKTIMLTGDNQATAQAIGQQVGVSEIEGELMPQDKL
CadA1_WRLP14    IVAVADEVRSSSQHVIKRLHELGIEKTIMLTGDNQATAQAIGQQVGVSEIEGELMPQDKL
CadA1_WRLP51    IVAVADEVRSSSQHVIKRLHELGIEKTIMLTGDNQATAQAIGQQVGVSEIEGELMPQDKL
CadA1_WRLP95    IVAVADEVRSSSQHVIKRLHELGIEKTIMLTGDNQATAQAIGQQVGVSEIEGELMPQDKL
CadA1_WRLP79    IVAVADEVRSSSQHVIKRLHELGIEKTIMLTGDNQATAQAIGQQVGVSEIEGELMPQDKL
CadA2_WRLP81    VIAVADEVRDSSAAVISELHKLSIEHTIMLTGDNTKTAESIGKQLGVTEIKGDLMPQEKL
CadA3_EGDe      IIAVADEVRESSKEVIAQLHKLGIAHTIMLTGDNNDTAQFIGKEIGVSDIKAELMPEDKL
CadA4_ScottA    SIVISDKIKKDSFEAIQSLKSKGVQKTVMLTGDNKVIAQNIAKELSLDEVYSELLPTDKV
                 :.::*::::..*  .*  *:. .: :*:******   *: *.:::.: :: .:*:* :*:

CadA1_WRLP46    DYIKQL---KINFGKVAMVGDGINDAPALAAATVGIAMGGAGTDTAIETADVALMGDDLQ
CadA1_WRLP76    DYIKQL---KINFGKVAMVGDGINDAPALAAATVGIAMGGAGTDTAIETADVALMGDDLQ
CadA1_WRLP22    DYIKQL---KINFGKVAMVGDGINDAPALAAATVGIAMGGAGTDTAIETADVALMGDDLQ
CadA1_WRLP13    DYIKQL---KINFGKVAMVGDGINDAPALAAATVGIAMGGAGTDTAIETADVALMGDDLQ
CadA1_WRLP14    DYIKQL---KINFGKVAMVGDGINDAPALAAATVGIAMGGAGTDTAIETADVALMGDDLQ
CadA1_WRLP51    DYIKQL---KINFGKVAMVGDGINDAPALAAATVGIAMGGAGTDTAIETADVALMGDDLQ
CadA1_WRLP95    DYIKQL---KINFGKVAMVGDGINDAPALAAATVGIAMGGAGTDTAIETADVALMGDDLQ
CadA1_WRLP79    DYIKQL---KINFGKVAMVGDGINDAPALAAATVGIAMGGAGTDTAIETADVALMGDDLQ
CadA2_WRLP81    DSIKAL---RTTYNKVAMVGDGINDAPALAASTVGIAMGGAGTDTALETADVALMGDDLQ
CadA3_EGDe      TYIKEL---KQTYGKVAMIGDGVNDAPALAASTVGIAMGGAGTDTALETADVALMGDDLK
CadA4_ScottA    DILERLENEEVDKGKLAFVGDGINDAPVLARADIGIAMGGLGSDAAIEAADIVLMTDEPS
                  :: *   .   .*:*::***:****.** : :****** *:*:*:*:**:.** *: .

CadA1_WRLP46    KLPFTVKLSRKTLQIIKQNITFSLVIKLIALLLVIPGWLTLWIAIMADMGATLLVTLNGL
CadA1_WRLP76    KLPFTVKLSRKTLQIIKQNITFSLVIKLIALLLVIPGWLTLWIAIMADMGATLLVTLNGL
CadA1_WRLP22    KLPFTVKLSRKTLQIIKQNITFSLVIKLIALLLVIPGWLTLWIAIMADMGATLLVTLNGL
CadA1_WRLP13    KLPFTVKLSRKTLQIIKQNITFSLVIKLIALLLVIPGWLTLWIAIMADMGATLLVTLNGL
CadA1_WRLP14    KLPFTVKLSRKTLQIIKQNITFSLVIKLIALLLVIPGWLTLWIAIMADMGATLLVTLNGL
CadA1_WRLP51    KLPFTVKLSRKTLQIIKQNITFSLVIKLIALLLVIPGWLTLWIAIMADMGATLLVTLNGL
CadA1_WRLP95    KLPFTVKLSRKTLQIIKQNITFSLVIKLIALLLVIPGWLTLWIAIMADMGATLLVTLNGL
CadA1_WRLP79    KLPFTVKLSRKTLQIIKQNITFSLVIKLIALLLVIPGWLTLWIAIMADMGATLLVTLNGL
CadA2_WRLP81    KLPFIVRLSRQTLKVIKQNITFSLGIKLLALLLVIPGWLTLWIAIVADMGATLLVTLNGL
CadA3_EGDe      KLPFIVNLSRKTLKIIKQNITFSLGIKLLALLLVLPGWLTLWIAIVADMGATLLVTLNGL
CadA4_ScottA    KIAKAIDISSFTKKIVWQNIIFALGVKAIFLTLGAFGIATMWEAVFADVGVSVLAILNAT
                *:.  : :*  * ::: *** *:* :* : * *   *  *:* *:.**:*.::*. **.
```

```
CadA1_WRLP46    RLMKVKD
CadA1_WRLP76    RLMKVKD
CadA1_WRLP22    RLMKVKD
CadA1_WRLP13    RLMKVKD
CadA1_WRLP14    RLMKVKD
CadA1_WRLP51    RLMKVKD
CadA1_WRLP95    RLMKVKD
CadA1_WRLP79    RLMKVKD
CadA2_WRLP81    RLMKVK-
CadA3_EGDe      RLMKVKK
CadA4_ScottA    RVIRYK-
                *::: *
```

Percent Identity Matrix

1: CadA1_WRLP46	100.00	100.00	100.00	100.00	100.00	100.00	100.00	100.00	69.47	71.27	36.83
2: CadA1_WRLP76	100.00	100.00	100.00	100.00	100.00	100.00	100.00	100.00	69.47	71.27	36.83
3: CadA1_WRLP22	100.00	100.00	100.00	100.00	100.00	100.00	100.00	100.00	69.47	71.27	36.83
4: CadA1_WRLP13	100.00	100.00	100.00	100.00	100.00	100.00	100.00	100.00	69.47	71.27	36.83
5: CadA1_WRLP14	100.00	100.00	100.00	100.00	100.00	100.00	100.00	100.00	69.47	71.27	36.83
6: CadA1_WRLP51	100.00	100.00	100.00	100.00	100.00	100.00	100.00	100.00	69.47	71.27	36.83
7: CadA1_WRLP95	100.00	100.00	100.00	100.00	100.00	100.00	100.00	100.00	69.47	71.27	36.83
8: CadA1_WRLP79	100.00	100.00	100.00	100.00	100.00	100.00	100.00	100.00	69.47	71.27	36.83
9: CadA2_WRLP81	69.47	69.47	69.47	69.47	69.47	69.47	69.47	69.47	100.00	73.76	37.54
10: CadA3_EGDe	71.27	71.27	71.27	71.27	71.27	71.27	71.27	71.27	73.76	100.00	37.48
11: CadA4_ScottA	36.83	36.83	36.83	36.83	36.83	36.83	36.83	36.83	37.54	37.48	100.00

Reference Sequence: CadC1_WRLP46

```
CadC1_WRLP46    MTVD--ICEITCIDEEKVKRVKTGL-ETVEVTTISQIFKILSDETRVKIVYALLTENELC
CadC1_WRLP13    MTVD--ICEITCIDEEKVKRVKTGL-ETVEVTTISQIFKILSDETRVKIVYALLTENELC
CadC1_WRLP14    MTVD--ICEITCIDEEKVKRVKTGL-ETVEVTTISQIFKILSDETRVKIVYALLTENELC
CadC1_WRLP95    MTVD--ICEITCIDEEKVKRVKTGL-ETVEVTTISQIFKILSDETRVKIVYALLTENELC
CadC1_WRLP22    MTVD--ICEITCIDEEKVKRVKTGL-ETVEVTTISQIFKILSDETRVKIVYALLTENELC
CadC1_WRLP51    MTVD--ICEITCIDEEKVKRVKTGL-ETVEVTTISQIFKILSDETRVKIVYALLTENELC
CadC1_WRLP79    MTVD--ICEITCIDEEKVKRVKTGL-ETVEVTTISQIFKILSDETRVKIVYALLTENELC
CadC1_WRLP76    MTVD--ICEITCIDEEKVKRVKTGL-ETVEVTTISQIFKILSDETRVKIVYALLTENELC
CadC3_EGDe      MND---ICEITCFDEEKVQKIQTSL-EDKKISDISQIFKALSEETRLTIAYALTLEKELC
CadC2_WRLP81    MNNE--ICEITCIHEDKVNRAKSKL-ANFDTPSVSGFFKILSDENRLKIVHALVHEDELC
CadC4_Scott     MNKQELICDCDVIHKEVVEKVKEVMPEKQAITNLTSLYKAFADKTRLEILYAL-HESEMC
                *.    **:   :.:: *:: :  :      . :: ::* ::::.*: * :**  *.*:*

CadC1_WRLP46    VCDLANIVEATVAATSHHLRFLKKQGIANYRKDGKLVYYSLANERVRDRIKLILLNF-EG
CadC1_WRLP13    VCDLANIVEATVAATSHHLRFLKKQGIANYRKDGKLVYYSLANERVRDRIKLILLNF-EG
CadC1_WRLP14    VCDLANIVEATVAATSHHLRFLKKQGIANYRKDGKLVYYSLANERVRDRIKLILLNF-EG
CadC1_WRLP95    VCDLANIVEATVAATSHHLRFLKKQGIANYRKDGKLVYYSLANERVRDRIKLILLNF-EG
CadC1_WRLP22    VCDLANIVEATVAATSHHLRFLKKQGIANYRKDGKLVYYSLANERVRDRIKLILLNF-EG
CadC1_WRLP51    VCDLANIVEATVAATSHHLRFLKKQGIANYRKDGKLVYYSLANERVRDRIKLILLNF-EG
CadC1_WRLP79    VCDLANIVEATVAATSHHLRFLKKQGIANYRKDGKLVYYSLANERVRDRIKLILLNF-EG
CadC1_WRLP76    VCDLANIVEATVAATSHHLRFLKKQGIANYRKDGKLVYYSLANERVRDRIKLILLNF-EG
CadC3_EGDe      VCDISIIVKSTVATTSHHLKILSKAGVVQNKKIGKMVYYSLNNSLVETLIK--------D
CadC2_WRLP81    VCDIANIIDASVATTSHHLNSLKKLGVVDSHKDGKLVYYFIKNIKILNLMELG-VNFKEE
CadC4_Scott     VCDLAVLLNMTKSAISHQLKTLRLANLVKNRREGKIVYYSLTDKHVYEIFE---QSF-KH
                ***:: ::. : :: **:*. *   .:.. :: **:*** : :  :   ::

CadC1_WRLP46    VGV
CadC1_WRLP13    VGV
CadC1_WRLP14    VGV
CadC1_WRLP95    VGV
CadC1_WRLP22    VGV
CadC1_WRLP51    VGV
CadC1_WRLP79    VGV
CadC1_WRLP76    VGV
CadC3_EGDe      IVL
CadC2_WRLP81    VLA
CadC4_Scott     VAE
                :
```

Percent Identity Matrix

1: CadC1_WRLP46	100.00	100.00	100.00	100.00	100.00	100.00	100.00	100.00	55.86	54.24	37.39
2: CadC1_WRLP13	100.00	100.00	100.00	100.00	100.00	100.00	100.00	100.00	55.86	54.24	37.39
3: CadC1_WRLP14	100.00	100.00	100.00	100.00	100.00	100.00	100.00	100.00	55.86	54.24	37.39
4: CadC1_WRLP95	100.00	100.00	100.00	100.00	100.00	100.00	100.00	100.00	55.86	54.24	37.39
5: CadC1_WRLP22	100.00	100.00	100.00	100.00	100.00	100.00	100.00	100.00	55.86	54.24	37.39
6: CadC1_WRLP51	100.00	100.00	100.00	100.00	100.00	100.00	100.00	100.00	55.86	54.24	37.39
7: CadC1_WRLP79	100.00	100.00	100.00	100.00	100.00	100.00	100.00	100.00	55.86	54.24	37.39
8: CadC1_WRLP76	100.00	100.00	100.00	100.00	100.00	100.00	100.00	100.00	55.86	54.24	37.39
9: CadC3_EGDe	55.86	55.86	55.86	55.86	55.86	55.86	55.86	55.86	100.00	47.75	37.27
10: CadC2_WRLP81	54.24	54.24	54.24	54.24	54.24	54.24	54.24	54.24	47.75	100.00	31.30
11: CadC4_Scott	37.39	37.39	37.39	37.39	37.39	37.39	37.39	37.39	37.27	31.30	100.00

Reference Sequence: 303aa_CzcD_WRLP46

```
303aa_CzcD_WRLP46     MAHNHDHAHGHNHNHAHNANKKSLFISFILIATFMVVEVIGGIMTNSLALLSDAGHMLSD
303aa_CzcD_WRLP85     MAHNHDHAHGHNHNHAHNANKKSLFISFILIATFMVVEVIGGIMTNSLALLSDAGHMLSD
303aa_CzcD_WRLP95     MAHNHDHAHGHNHNHAHNANKKSLFISFILIATFMVVEVIGGIMTNSLALLSDAGHMLSD
303aa_CzcD_EGDe       MAHNHDHAHGHNHNHAHNANKKSLFISFILIATFMVVEVIGGIMTNSLALLSDAGHMLSD
303aa_CzcD_WRLP81     MAHNHDHAHGHNHNHAHNANKKSLFISFILIATFMVVEVIGGIMTNSLALLSDAGHMLSD
303aa_CzcD_ScottA     MAHNHDHAHGHNHNHAHNANKKSLFISFILIATFMVVEVIGGIMTNSLALLSDAGHMLSD
                      ************************************************************

303aa_CzcD_WRLP46     AVALGLSLAAFKFGEKAASSDKTYGYKRFEILAAFLNGLTLVGISVFIFYEAIGRFFDPP
303aa_CzcD_WRLP85     AVALGLSLAAFKFGEKAASSDKTYGYKRFEILAAFLNGLTLVGISVFIFYEAIGRFFDPP
303aa_CzcD_WRLP95     AVALGLSLAAFKFGEKAASSDKTYGYKRFEILAAFLNGLTLVGISVFIFYEAIGRFFDPP
303aa_CzcD_EGDe       AVALGLSLAAFKFGEKAASSDKTYGYKRFEILAAFLNGLTLVGISVFIFYEAIGRFFDPP
303aa_CzcD_WRLP81     AVALGLSLAAFKFGEKAASSDKTYGYKRFEILAAFLNGLTLVGISVFIFYEAIGRFFDPP
303aa_CzcD_ScottA     AVALGLSLAAFKFGEKAASSDKTYGYKRFEILAAFLNGLTLVGISVFIFYEAIGRFFDPP
                      ************************************************************

303aa_CzcD_WRLP46     QVIGAGMMTISVIGLLINILVAWILMKGDTSENLNMRSAFLHVLGDLLGSVGAIIAALLI
303aa_CzcD_WRLP85     QVIGAGMMTISVIGLLINILVAWILMKGDTSENLNMRSAFLHVLGDLLGSVGAIIAALLI
303aa_CzcD_WRLP95     QVIGAGMMTISVIGLLINILVAWILMKGDTSENLNMRSAFLHVLGDLLGSVGAIIAALLI
303aa_CzcD_EGDe       QVIGAGMMTISVIGLLINILVAWILMKGDTSENLNMRSAFLHVLGDLLGSVGAIIAALLI
303aa_CzcD_WRLP81     QVIGAGMMTISVIGLLINILVAWILMKGDTSENLNMRSAFLHVLGDLLGSVGAIIAALLI
303aa_CzcD_ScottA     QVIGAGMMTISVIGLLINILVAWILMKGDTSENLNMRSAFLHVLGDLLGSVGAIIAALLI
                      ************************************************************

303aa_CzcD_WRLP46     IFLGWNIADPIASVIVAALILVSGWRVLKDAIHILMEGKPANVDTEEIKTFFQQQDGVKE
303aa_CzcD_WRLP85     IFLGWNIADPIASVIVAALILVSGWRVLKDAIHILMEGKPANVDTEEIKTFFQQQDGVKE
303aa_CzcD_WRLP95     IFLGWNIADPIASVIVAALILVSGWRVLKDAIHILMEGKPANVDTEEIKTFFQQQDGVKE
303aa_CzcD_EGDe       IFLGWNIADPIASVIVAALILVSGWRVLKDAIHILMEGKPANVDTEEIKTFFQQQDGVKE
303aa_CzcD_WRLP81     IFLGWNIADPIASVIVAALILVSGWRVLKDAIHILMEGKPANVDTEEIKTFFQQQDGVKE
303aa_CzcD_ScottA     IFLGWNIADPIASVIVAALILVSGWRVLKDAIHILMEGKPANVDTEEIKTFFQQQDGVKE
                      ************************************************************

303aa_CzcD_WRLP46     VHDLHVWAITSDFNALSAHLTVCEDADRDKILADIEHYLQENFSLEHSTIQLEGNHAHHH
303aa_CzcD_WRLP85     VHDLHVWAITSDFNALSAHLTVCEDADRDKILADIEHYLQENFSLEHSTIQLEGNHAHHH
303aa_CzcD_WRLP95     VHDLHVWAITSDFNALSAHLTVCEDADRDKILADIEHYLQENFSLEHSTIQLEGNHAHHH
303aa_CzcD_EGDe       VHDLHVWAITSDFNALSAHLTVCEDADRDKILADIEHYLQENFSLEHSTIQLEGNHAHHH
303aa_CzcD_WRLP81     VHDLHVWAITSDFNALTAHLTVCEDADRDKILADIEHYLQENFSLEHSTIQLEGNHAHHH
303aa_CzcD_ScottA     VHDLHVWAITSDFNALTAHLTVCEDADRDKILADIEHYLQENFSLEHSTIQLEGNHAHHH
                      ****************:*******************************************

303aa_CzcD_WRLP46     HCN
303aa_CzcD_WRLP85     HCN
303aa_CzcD_WRLP95     HCN
303aa_CzcD_EGDe       HCN
303aa_CzcD_WRLP81     HCN
303aa_CzcD_ScottA     HCN
                      ***

Percent Identity  Matrix
     1: 303aa_CzcD_WRLP46  100.00  100.00  100.00  100.00   99.67   99.67
     2: 303aa_CzcD_WRLP85  100.00  100.00  100.00  100.00   99.67   99.67
     3: 303aa_CzcD_WRLP95  100.00  100.00  100.00  100.00   99.67   99.67
     4: 303aa_CzcD_EGDe    100.00  100.00  100.00  100.00   99.67   99.67
     5: 303aa_CzcD_WRLP81   99.67   99.67   99.67   99.67  100.00  100.00
     6: 303aa_CzcD_ScottA   99.67   99.67   99.67   99.67  100.00  100.00
```
