# Supplementary material for: Variability in cadmium tolerance of closely related Listeria monocytogenes isolates originating from dairy processing environments
Source: Appl Environ Microbiol. 2024 Nov 21;91(1):e01281-24. doi: 10.1128/aem.01281-24 (PMC11784300; doi:10.1128/aem.01281-24)
Supplement: Tables S1 and S2 — Background information on L. monocytogenes isolates used in the study and lag phase duration for cadA+ isolates when exposed to cadmium at 8 ppm. [file aem.01281-24-s0002.docx]

**Supplemental Materials**

**Table S1** Background information on *Listeria monocytogenes* isolates used in this study.

| **General Information** | | | | | | **Classification** | | | | | | **AMR Related Genes** | | |
| --- | --- | --- | --- | --- | --- | --- | --- | --- | --- | --- | --- | --- | --- | --- |
| **Isolate ID** | **Year** | **Facility** | **Clonal group membership** | **Source Detail** | **Additional Source Detail** | **Lineage** | **MLST (ST) - Pasteur** | **CC - Pasteur** | **SL** | ***sigB*** | **CT** | **Sanitizer efflux genes** | **Fluroquinolone efflux** | **Heavy Metal Genes** |
| WRLP7 | 2009 | 71 | FALSE | Environmental | Floor Drain | 2 | 7 | 7 | 7 | 1 | 10086 |  | *fepA, lde, mdrL* |  |
| WRLP8 | 2009 | 71 | FALSE | Environmental | Floor Drain | 2 | 7 | 7 | 7 | 1 | 10086 |  | *fepA, lde, mdrL* |  |
| WRLP9 | 2009 | 71 | FALSE | Environmental | Floor Drain | 2 | 7 | 7 | 7 | 1 | 10086 |  | *fepA, lde, mdrL* |  |
| WRLP10 | 2009 | 71 | TRUE | Environmental | Cinder Bucket Under Wash Sink | 2 | 11 | 11 | 11 | 6 | 6558 |  | *fepA, lde, mdrL* | *cadA1* |
| WRLP11 | 2009 | 71 | TRUE | Environmental | Cinder Bucket Under Wash Sink | 2 | 11 | 11 | 11 | 6 | 6558 |  | *fepA, lde, mdrL* | *cadA1* |
| WRLP12 | 2009 | 71 | TRUE | Environmental | Cinder Bucket Under Wash Sink | 2 | 11 | 11 | 11 | 6 | 6558 |  | *fepA, lde, mdrL* | *cadA1* |
| WRLP13 | 2009 | 71 | TRUE | Environmental | Draining Rack | 2 | 11 | 11 | 11 | 6 | 6558 |  | *fepA, lde, mdrL* | *cadA1* |
| WRLP14 | 2009 | 71 | TRUE | Environmental | Draining Rack | 2 | 11 | 11 | 11 | 6 | 6558 |  | *fepA, lde, mdrL* | *cadA1* |
| WRLP15 | 2009 | 71 | TRUE | Environmental | Draining Rack | 2 | 11 | 11 | 11 | 6 | 6558 |  | *fepA, lde, mdrL* | *cadA1* |
| WRLP16 | 2007 | 71 | TRUE | Environmental | Cooler Drain | 2 | 11 | 11 | 11 | 6 | 6558 |  | *fepA, lde, mdrL* | *cadA1* |
| WRLP17 | 2007 | 71 | TRUE | Environmental | Main Floor Drain | 2 | 11 | 11 | 11 | 6 | 6558 |  | *fepA, lde, mdrL* | *cadA1* |
| WRLP18 | 2010 | 71 | TRUE | Environmental | Drain (Camembert Room Drain) | 2 | 11 | 11 | 11 | 6 | 6558 |  | *fepA, lde, mdrL* | *cadA1* |
| WRLP19 | 2010 | 71 | TRUE | Environmental | Drain Table, Underside Of Spout | 2 | 11 | 11 | 11 | 6 | 6558 |  | *fepA, lde, mdrL* | *cadA1* |
| WRLP20 | 2010 | 71 | TRUE | Environmental | Drain (By Drain Table) | 2 | 11 | 11 | 11 | 6 | 6558 |  | *fepA, lde, mdrL* | *cadA1* |
| WRLP21 | 2010 | 71 | TRUE | Environmental | Drain (Enterance) | 2 | 11 | 11 | 11 | 6 | 6558 |  | *fepA, lde, mdrL* | *cadA1* |
| WRLP22 | 2010 | 71 | TRUE | Environmental | Drain (Cooler) | 2 | 11 | 11 | 11 | 6 | 6558 |  | *fepA, lde, mdrL* | *cadA1* |
| WRLP23 | 2010 | 71 | TRUE | Environmental | Drain (Blue Room) | 2 | 11 | 11 | 11 | 6 | 6558 |  | *fepA, lde, mdrL* | *cadA1* |
| WRLP24 | 2010 | 71 | TRUE | Environmental | Drain (Tomme) | 2 | 11 | 11 | 11 | 6 | 6558 |  | *fepA, lde, mdrL* | *cadA1* |
| WRLP26 | 2012 | 71 | TRUE | Food | Tomme D'Or Cheese | 2 | 11 | 11 | 11 | 6 | 6558 |  | *fepA, lde, mdrL* | *cadA1* |
| WRLP27 | 2012 | 71 | TRUE | Food | Tomme D'Or Cheese | 2 | 11 | 11 | 11 | 6 | 6558 |  | *fepA, lde, mdrL* | *cadA1* |
| WRLP28 | 2012 | 71 | TRUE | Food | Tomme D'Or Cheese | 2 | 11 | 11 | 11 | 6 | 6558 |  | *fepA, lde, mdrL* | *cadA1* |
| WRLP29 | 2012 | 71 | TRUE | Food | Tomme D'Or Cheese | 2 | 11 | 11 | 11 | 6 | 6558 |  | *fepA, lde, mdrL* | *cadA1* |
| WRLP30 | 2012 | 71 | TRUE | Food | Tomme D'Or Cheese | 2 | 11 | 11 | 11 | 6 | 6558 |  | *fepA, lde, mdrL* | *cadA1* |
| WRLP31 | 2012 | 71 | TRUE | Food | Tomme D'Or Cheese | 2 | 11 | 11 | 11 | 6 | 6558 |  | *fepA, lde, mdrL* | *cadA1* |
| WRLP32 | 2012 | 71 | TRUE | Food | Tomme D'Or Cheese | 2 | 11 | 11 | 11 | 6 | 6558 |  | *fepA, lde, mdrL* | *cadA1* |
| WRLP33 | 2012 | 71 | TRUE | Food | Tomme D'Or Cheese | 2 | 11 | 11 | 11 | 6 | 6558 |  | *fepA, lde, mdrL* | *cadA1* |
| WRLP34 | 2012 | 71 | TRUE | Food | Tomme D'Or Cheese | 2 | 11 | 11 | 11 | 6 | 6558 |  | *fepA, lde, mdrL* | *cadA1* |
| WRLP35 | 2012 | 71 | TRUE | Food | Tomme D'Or Cheese | 2 | 11 | 11 | 11 | 6 | 6558 |  | *fepA, lde, mdrL* | *cadA1* |
| WRLP36 | 2012 | 71 | TRUE | Food | Tomme D'Or Cheese | 2 | 11 | 11 | 11 | 6 | 6558 |  | *fepA, lde, mdrL* | *cadA1* |
| WRLP37 | 2012 | 71 | TRUE | Food | Tomme D'Or Cheese | 2 | 11 | 11 | 11 | 6 | 6558 |  | *fepA, lde, mdrL* | *cadA1* |
| WRLP38 | 2012 | 71 | TRUE | Food | Tomme D'Or Cheese | 2 | 11 | 11 | 11 | 6 | 6558 |  | *fepA, lde, mdrL* | *cadA1* |
| WRLP39 | 2012 | 71 | TRUE | Food | Tomme D'Or Cheese | 2 | 11 | 11 | 11 | 6 | 6558 |  | *fepA, lde, mdrL* | *cadA1* |
| WRLP40 | 2012 | 71 | TRUE | Food | Tomme D'Or Cheese | 2 | 11 | 11 | 11 | 6 | 6558 |  | *fepA, lde, mdrL* | *cadA1* |
| WRLP41 | 2012 | 71 | TRUE | Food | Tomme D'Or Cheese | 2 | 11 | 11 | 11 | 6 | 6558 |  | *fepA, lde, mdrL* | *cadA1* |
| WRLP42 | 2012 | 71 | TRUE | Food | Tomme D'Or Cheese | 2 | 11 | 11 | 11 | 6 | 6558 |  | *fepA, lde, mdrL* | *cadA1* |
| WRLP43 | 2012 | 71 | TRUE | Food | Tomme D'Or Cheese | 2 | 11 | 11 | 11 | 6 | 6558 |  | *fepA, lde, mdrL* | *cadA1* |
| WRLP44 | 2012 | 71 | TRUE | Food | Tomme D'Or Cheese | 2 | 11 | 11 | 11 | 6 | 6558 |  | *fepA, lde, mdrL* | *cadA1* |
| WRLP45 | 2012 | 71 | TRUE | Food | Tomme D'Or Cheese | 2 | 11 | 11 | 11 | 6 | 6558 |  | *fepA, lde, mdrL* | *cadA1* |
| WRLP46 | 2012 | 71 | TRUE | Food | Tomme D'Or Cheese | 2 | 11 | 11 | 11 | 6 | 6558 |  | *fepA, lde, mdrL* | *cadA1* |
| WRLP47 | 2012 | 71 | TRUE | Food | Tomme D'Or Cheese | 2 | 11 | 11 | 11 | 6 | 6558 |  | *fepA, lde, mdrL* | *cadA1* |
| WRLP48 | 2012 | 71 | TRUE | Food | Tomme D'Or Cheese | 2 | 11 | 11 | 11 | 6 | 6558 |  | *fepA, lde, mdrL* | *cadA1* |
| WRLP49 | 2012 | 71 | TRUE | Food | Tomme D'Or Cheese | 2 | 11 | 11 | 11 | 6 | 6558 |  | *fepA, lde, mdrL* | *cadA1* |
| WRLP50 | 2012 | 71 | TRUE | Food | Tomme D'Or Cheese | 2 | 11 | 11 | 11 | 6 | 6558 |  | *fepA, lde, mdrL* | *cadA1* |
| WRLP51 | 2012 | 71 | TRUE | Food | Tomme D'Or Cheese | 2 | 11 | 11 | 11 | 6 | 6558 |  | *fepA, lde, mdrL* | *cadA1* |
| WRLP52 | 2012 | 71 | TRUE | Food | Tomme D'Or Cheese | 2 | 11 | 11 | 11 | 6 | 6558 |  | *fepA, lde, mdrL* | *cadA1* |
| WRLP53 | 2012 | 71 | TRUE | Food | Tomme D'Or Cheese | 2 | 11 | 11 | 11 | 6 | 6558 |  | *fepA, lde, mdrL* | *cadA1* |
| WRLP54 | 2012 | 71 | TRUE | Food | Tomme D'Or Cheese | 2 | 11 | 11 | 11 | 6 | 6558 |  | *fepA, lde, mdrL* | *cadA1* |
| WRLP55 | 2012 | 71 | TRUE | Food | Tomme D'Or Cheese | 2 | 11 | 11 | 11 | 6 | 6558 |  | *fepA, lde, mdrL* | *cadA1* |
| WRLP56 | 2012 | 71 | TRUE | Food | Tomme D'Or Cheese | 2 | 11 | 11 | 11 | 6 | 6558 |  | *fepA, lde, mdrL* | *cadA1* |
| WRLP57 | 2012 | 71 | TRUE | Food | Tomme D'Or Cheese | 2 | 11 | 11 | 11 | 6 | 6558 |  | *fepA, lde, mdrL* | *cadA1* |
| WRLP58 | 2012 | 71 | TRUE | Food | Tomme D'Or Cheese | 2 | 11 | 11 | 11 | 6 | 6558 |  | *fepA, lde, mdrL* | *cadA1* |
| WRLP59 | 2012 | 71 | TRUE | Food | Tomme D'Or Cheese | 2 | 11 | 11 | 11 | 6 | 6558 |  | *fepA, lde, mdrL* | *cadA1* |
| WRLP60 | 2012 | 71 | TRUE | Food | Tomme D'Or Cheese | 2 | 11 | 11 | 11 | 6 | 6558 |  | *fepA, lde, mdrL* | *cadA1* |
| WRLP61 | 2012 | 71 | TRUE | Food | Tomme D'Or Cheese | 2 | 11 | 11 | 11 | 6 | 6558 |  | *fepA, lde, mdrL* | *cadA1* |
| WRLP62 | 2012 | 71 | TRUE | Food | Tomme D'Or Cheese | 2 | 11 | 11 | 11 | 6 | 6558 |  | *fepA, lde, mdrL* | *cadA1* |
| WRLP63 | 2012 | 71 | TRUE | Food | Tomme D'Or Cheese | 2 | 11 | 11 | 11 | 6 | 6558 |  | *fepA, lde, mdrL* | *cadA1* |
| WRLP64 | 2012 | 71 | TRUE | Food | Tomme D'Or Cheese | 2 | 11 | 11 | 11 | 6 | 6558 |  | *fepA, lde, mdrL* | *cadA1* |
| WRLP65 | 2012 | 71 | TRUE | Food | Tomme D'Or Cheese | 2 | 11 | 11 | 11 | 6 | 6558 |  | *fepA, lde, mdrL* | *cadA1* |
| WRLP66 | 2012 | 71 | TRUE | Food | Tomme D'Or Cheese | 2 | 11 | 11 | 11 | 6 | 6558 |  | *fepA, lde, mdrL* | *cadA1* |
| WRLP67 | 2012 | 71 | TRUE | Food | Tomme D'Or Cheese | 2 | 11 | 11 | 11 | 6 | 6558 |  | *fepA, lde, mdrL* | *cadA1* |
| WRLP68 | 2012 | 71 | TRUE | Food | Tomme D'Or Cheese | 2 | 11 | 11 | 11 | 6 | 6558 |  | *fepA, lde, mdrL* | *cadA1* |
| WRLP69 | 2012 | 71 | TRUE | Environmental | Raw Milk Pump Shaft | 2 | 11 | 11 | 11 | 6 | 6558 |  | *fepA, lde, mdrL* | *cadA1* |
| WRLP70 | 2012 | 71 | FALSE | Food | Blossom Blue Cheese | 1 | 224 | 224 | 11 | 13 | 6679 |  | *fepA, lde, mdrL* | *cadA1* |
| WRLP71 | 2015 | 71 | TRUE | Environmental | Aging Room Drain | 2 | 11 | 11 | 11 | 6 | 6558 |  | *fepA, lde, mdrL* | *cadA1* |
| WRLP73 | 2015 | 71 | FALSE | Food | Blue Cheese Variety 1 | 1 | 1 | 1 | 1 | 2 | 6441 |  | *fepA, lde, mdrL* |  |
| WRLP74 | 2015 | 71 | FALSE | Food | Blue Cheese Variety 2 | 1 | 397 | 4 | 397 | 4 | 6720 |  | *fepA, lde, mdrL* |  |
| WRLP75 | 2016 | 71 | TRUE | Environmental | Brine Room Cartwheels | 2 | 11 | 11 | 11 | 6 | 6558 |  | *fepA, lde, mdrL* | *cadA1* |
| WRLP76 | 2016 | 71 | FALSE | Environmental | Aging Room Door Sweep | 2 | 11 | 11 | 11 | 6 | 6558 |  | *fepA, lde, mdrL* | *cadA1* |
| WRLP77 | 2016 | 71 | TRUE | Environmental | Aging Room #1 Drain | 2 | 11 | 11 | 11 | 6 | 6558 |  | *fepA, lde, mdrL* | *cadA1* |
| WRLP78 | 2016 | 71 | TRUE | Environmental | Aging Room #3 Drain | 2 | 11 | 11 | 11 | 6 | 6558 |  | *fepA, lde, mdrL* | *cadA1* |
| WRLP79 | 2016 | 71 | FALSE | Environmental | Drain Table Wheel | 2 | 11 | 11 | 11 | 6 | 6558 |  | *fepA, lde, mdrL* | *cadA1* |
| WRLP80 | 2016 | 71 | FALSE | Environmental | Raw Milk Pump Frame | 1 | 1 | 1 | 1 | 2 | 252 |  | *fepA, lde, mdrL* | *cadA1* |
| WRLP81 | 2015 | 106 | FALSE | Environmental | Process Room Drain | 1 | 288 | 288 | 288 | 3 | 6544 |  | *fepA, lde, mdrL* | *cadA2* |
| WRLP82 | 2017 | 122 | FALSE | Environmental | Cheese Wash Sink | 2 | 399 | 14 | 399 | 6 | 10088 |  | *fepA, lde, mdrL* | *cadA1* |
| WRLP83 | 2017 | 122 | FALSE | Environmental | Aging Room Shelf | 2 | 399 | 14 | 399 | 6 | 10088 |  | *fepA, lde, mdrL* | *cadA1* |
| WRLP84 | 2017 | 122 | FALSE | Food | Wash Rind Cheese | 2 | 399 | 14 | 399 | 6 | 10088 |  | *fepA, lde, mdrL* |  |
| WRLP85 | 2017 | 122 | FALSE | Food | Wash Rind Cheese | 2 | 399 | 14 | 399 | 6 | 10088 |  | *fepA, lde, mdrL* | *cadA1* |
| WRLP86 | 2017 | 122 | FALSE | Food | Wash Rind Cheese | 2 | 399 | 14 | 399 | 6 | 10088 |  | *fepA, lde, mdrL* |  |
| WRLP87 | 2017 | 122 | FALSE | Food | Wash Rind Cheese | 2 | 399 | 14 | 399 | 6 | 10088 |  | *fepA, lde, mdrL* |  |
| WRLP88 | 2017 | 122 | FALSE | Environmental | Cheese Wash Sink Draining Bucket | 2 | 399 | 14 | 399 | 6 | 10088 |  | *fepA, lde, mdrL* |  |
| WRLP89 | 2017 | 122 | FALSE | Environmental | Floor Near Drain | 2 | 399 | 14 | 399 | 6 | 10088 |  | *fepA, lde, mdrL* |  |
| WRLP90 | 2017 | 122 | FALSE | Food | Wash Rind Cheese | 2 | 399 | 14 | 399 | 6 | 10088 |  | *fepA, lde, mdrL* |  |
| WRLP91 | 2017 | 122 | FALSE | Food | Wash Rind Cheese | 2 | 399 | 14 | 399 | 6 | 10088 |  | *fepA, lde, mdrL* |  |
| WRLP92 | 2017 | 122 | FALSE | Food | Wash Rind Cheese | 2 | 399 | 14 | 399 | 6 | 10088 |  | *fepA, lde, mdrL* |  |
| WRLP93 | 2017 | 122 | FALSE | Food | Wash Rind Cheese | 2 | 399 | 14 | 399 | 6 | 10088 |  | *fepA, lde, mdrL* |  |
| WRLP94 | 2016 | 14 | FALSE | Environmental | Case Room Floor | 2 | 2803 | 226 | 226 | 1 | 10475 |  | *fepA, lde, mdrL* |  |
| WRLP95 | 2017 | 14 | FALSE | Environmental | Floor Under Cap Sealer | 2 | 11 | 11 | 11 | 6 | 10087 | *bcrABC* | *fepA, lde, mdrL* | *cadA1* |
| WRLP96 | 2016 | 131 | FALSE | Environmental | Kitchen Floor Near Sink | 1 | 6 | 6 | 6 | 4 | 10089 |  | *fepA, lde, mdrL* |  |

**Table S2** Increase in lag phase duration (LPD; h) for *cadA*^+^ isolates when exposed to cadmium at 8 ppm (43.8 μM) CdCl_2_.

| **Isolate ID** | **LPD±SD (h) with cadmium** | **LPD±SD (h) without cadmium** | **Increase in LPD (h)** | **Facility** | **Facility 71 clonal cluster member** |
| --- | --- | --- | --- | --- | --- |
| WRLP10 | 10.94±0.12 | 8.69±0.01 | 2.25±0.13 | 71 | TRUE |
| WRLP11 | 10.99±0.17 | 7.84±0.01 | 3.15±0.17 | 71 | TRUE |
| WRLP12 | 11.75±0.08 | 8.38±0.32 | 3.37±0.31 | 71 | TRUE |
| WRLP13 | 12.88±0.25 | 8.43±0.31 | 4.45±0.18 | 71 | TRUE |
| WRLP14 | 12.89±0.38 | 8.33±0.13 | 4.56±0.49 | 71 | TRUE |
| WRLP15 | 10.11±0.12 | 7.81±0.13 | 2.30±0.18 | 71 | TRUE |
| WRLP16 | 11.06±0.17 | 8.10±0.13 | 2.97±0.30 | 71 | TRUE |
| WRLP17 | 11.13±0.03 | 8.57±0.13 | 2.56±0.14 | 71 | TRUE |
| WRLP18 | 8.97±0.04 | 7.09±0.10 | 1.88±0.09 | 71 | TRUE |
| WRLP19 | 11.13±0.28 | 8.43±0.16 | 2.70±0.43 | 71 | TRUE |
| WRLP20 | 9.03±0.13 | 6.74±0.05 | 2.30±0.14 | 71 | TRUE |
| WRLP21 | 11.50±0.43 | 8.62±0.03 | 2.88±0.45 | 71 | TRUE |
| WRLP22 | 7.03±0.13 | 6.04±0.04 | 0.99±0.14 | 71 | TRUE |
| WRLP23 | 10.75±0.08 | 7.64±0.22 | 3.11±0.21 | 71 | TRUE |
| WRLP24 | 11.26±0.08 | 8.54±0.19 | 2.72±0.22 | 71 | TRUE |
| WRLP26 | 10.89±0.06 | 8.58±0.41 | 2.31±0.47 | 71 | TRUE |
| WRLP27 | 10.99±0.22 | 8.46±0.15 | 2.53±0.12 | 71 | TRUE |
| WRLP28 | 11.36±0.17 | 8.50±0.17 | 2.85±0.17 | 71 | TRUE |
| WRLP29 | 11.73±0.23 | 8.49±0.25 | 3.24±0.44 | 71 | TRUE |
| WRLP30 | 11.46±0.23 | 8.33±0.07 | 3.13±0.30 | 71 | TRUE |
| WRLP31 | 10.30±0.08 | 7.07±0.14 | 3.23±0.09 | 71 | TRUE |
| WRLP32 | 11.47±0.22 | 8.41±0.16 | 3.06±0.38 | 71 | TRUE |
| WRLP33 | 9.99±0.14 | 6.88±0.09 | 3.11±0.22 | 71 | TRUE |
| WRLP34 | 11.15±0.04 | 8.39±0.07 | 2.75±0.03 | 71 | TRUE |
| WRLP35 | 11.08±0.29 | 8.19±0.04 | 2.89±0.26 | 71 | TRUE |
| WRLP36 | 11.15±0.13 | 8.30±0.05 | 2.85±0.12 | 71 | TRUE |
| WRLP37 | 11.14±0.18 | 8.29±0.20 | 2.85±0.11 | 71 | TRUE |
| WRLP38 | 11.18±0.19 | 8.09±0.16 | 3.09±0.34 | 71 | TRUE |
| WRLP39 | 10.39±0.21 | 7.95±0.23 | 2.44±0.38 | 71 | TRUE |
| WRLP40 | 11.59±0.32 | 9.41±0.26 | 2.18±0.06 | 71 | TRUE |
| WRLP41 | 10.51±0.30 | 8.09±0.02 | 2.42±0.28 | 71 | TRUE |
| WRLP42 | 11.76±0.06 | 9.57±0.05 | 2.19±0.01 | 71 | TRUE |
| WRLP43 | 11.41±0.12 | 9.50±0.15 | 1.91±0.24 | 71 | TRUE |
| WRLP44 | 11.80±0.29 | 9.71±0.06 | 2.09±0.26 | 71 | TRUE |
| WRLP45 | 12.03±0.16 | 9.74±0.15 | 2.30±0.21 | 71 | TRUE |
| WRLP46 | 12.30±0.20 | 9.75±0.19 | 2.55±0.20 | 71 | TRUE |
| WRLP47 | 11.80±0.31 | 9.77±0.19 | 2.03±0.32 | 71 | TRUE |
| WRLP48 | 11.96±0.13 | 9.71±0.01 | 2.25±0.12 | 71 | TRUE |
| WRLP49 | 12.11±0.48 | 9.46±0.07 | 2.65±0.55 | 71 | TRUE |
| WRLP50 | 11.22±0.12 | 9.49±0.06 | 1.73±0.07 | 71 | TRUE |
| WRLP51 | 10.49±0.19 | 9.13±0.08 | 1.35±0.21 | 71 | TRUE |
| WRLP52 | 10.81±0.26 | 7.74±0.23 | 3.07±0.48 | 71 | TRUE |
| WRLP53 | 11.41±0.06 | 9.35±0.05 | 2.05±0.03 | 71 | TRUE |
| WRLP54 | 11.06±0.29 | 9.44±0.19 | 1.62±0.37 | 71 | TRUE |
| WRLP55 | 10.87±0.04 | 8.81±0.26 | 2.06±0.22 | 71 | TRUE |
| WRLP56 | 11.93±0.19 | 9.21±0.08 | 2.71±0.19 | 71 | TRUE |
| WRLP57 | 12.08±0.02 | 9.27±0.10 | 2.81±0.08 | 71 | TRUE |
| WRLP58 | 11.60±0.15 | 8.83±0.21 | 2.77±0.26 | 71 | TRUE |
| WRLP59 | 11.06±0.15 | 8.59±0.01 | 2.47±0.16 | 71 | TRUE |
| WRLP60 | 12.20±0.27 | 9.01±0.22 | 3.19±0.15 | 71 | TRUE |
| WRLP61 | 12.77±0.21 | 9.05±0.02 | 3.72±0.20 | 71 | TRUE |
| WRLP62 | 12.03±0.07 | 8.05±0.31 | 3.99±0.26 | 71 | TRUE |
| WRLP63 | 11.58±0.36 | 8.18±0.02 | 3.40±0.35 | 71 | TRUE |
| WRLP64 | 11.50±0.34 | 8.32±0.17 | 3.18±0.46 | 71 | TRUE |
| WRLP65 | 12.27±0.28 | 8.75±0.12 | 3.52±0.32 | 71 | TRUE |
| WRLP66 | 11.96±0.60 | 9.51±0.19 | 2.45±0.42 | 71 | TRUE |
| WRLP67 | 11.79±0.34 | 9.56±0.05 | 2.23±0.38 | 71 | TRUE |
| WRLP68 | 11.74±0.26 | 9.32±0.11 | 2.42±0.24 | 71 | TRUE |
| WRLP69 | 11.98±0.56 | 9.00±0.05 | 2.98±0.54 | 71 | TRUE |
| WRLP71 | 10.26±0.26 | 8.10±0.13 | 2.16±0.12 | 71 | TRUE |
| WRLP75 | 10.71±0.06 | 8.43±0.33 | 2.28±0.36 | 71 | FALSE |
| WRLP76 | 10.30±0.09 | 8.33±0.06 | 1.96±0.15 | 71 | TRUE |
| WRLP77 | 11.01±0.29 | 8.26±0.11 | 2.75±0.21 | 71 | TRUE |
| WRLP78 | 12.14±0.10 | 8.81±0.31 | 3.32±0.40 | 71 | TRUE |
| WRLP79 | 11.63±0.07 | 9.01±0.33 | 2.62±0.40 | 71 | FALSE |
| WRLP81 | 9.03±0.22 | 7.71±0.04 | 1.32±0.17 | 106 | FALSE |
| WRLP95 | 14.82±0.18 | 8.38±0.13 | 6.44±0.08 | 14 | FALSE |
